# Pulmonary function and atherosclerosis in the general population: causal associations and clinical implications

**DOI:** 10.1007/s10654-023-01088-z

**Published:** 2024-01-02

**Authors:** Gunnar Engström, Erik Lampa, Koen Dekkers, Yi-Ting Lin, Kristin Ahlm, Håkan Ahlström, Joakim Alfredsson, Göran Bergström, Anders Blomberg, John Brandberg, Kenneth Caidahl, Kerstin Cederlund, Olov Duvernoy, Jan E. Engvall, Maria J. Eriksson, Tove Fall, Bruna Gigante, Anders Gummesson, Emil Hagström, Viktor Hamrefors, Jan Hedner, Magnus Janzon, Tomas Jernberg, Linda Johnson, Lars Lind, Eva Lindberg, Maria Mannila, Ulf Nilsson, Anders Persson, Hans Lennart Persson, Margaretha Persson, Anna Ramnemark, Annika Rosengren, Caroline Schmidt, Linn Skoglund Larsson, C. Magnus Sköld, Eva Swahn, Stefan Söderberg, Kjell Torén, Anders Waldenström, Per Wollmer, Suneela Zaigham, Carl Johan Östgren, Johan Sundström

**Affiliations:** 1 Department of Clinical Sciences in Malmö, Lund University, Lund, Sweden; 2 Department of Medical Sciences, Uppsala University, Uppsala, Sweden; 3 Department of Medical Sciences, Molecular Epidemiology and Science for Life Laboratory, Uppsala University, Uppsala, Sweden; 4 Department of Neurobiology, Care Sciences and Society, Karolinska Institute, Huddinge, Sweden; 5 Department of Family Medicine, Kaohsiung Medical University, Kaohsiung City, Taiwan; 6 Department of Public Health and Clinical Medicine, Section of Medicine, Umeå University, Umeå, Sweden; 7 Department of Surgical Sciences, Section of Radiology, Uppsala University, Uppsala, Sweden; 8 BFC, Uppsala University Hospital, Uppsala, Sweden; 9 Antaros Medical AB, Mölndal, Sweden; 10 Department of Cardiology, Department of Health, Medicine and Caring Sciences, Unit of Cardiovascular Sciences, Linköping University, Linköping, Sweden; 11 Department of Molecular and Clinical Medicine, Institute of Medicine, Sahlgrenska Academy, University of Gothenburg, Gothenburg, Sweden; 12 Clinical Physiology, Sahlgrenska University Hospital, Gothenburg, Sweden; 13 Department of Radiology, Region Västra Götaland, Sahlgrenska University Hospital, Gothenburg, Sweden; 14 Department of Radiology, Institute of Clinical Sciences, Sahlgrenska Academy, University of Gothenburg, Gothenburg, Sweden; 15 Department of Clinical Physiology, Karolinska University Hospital, Stockholm, Sweden; 16 Department of Clinical Physiology, Sahlgrenska University Hospital, Sahlgrenska Academy, Gothenburg, Sweden; 17 Department of Clinical Science, Intervention and Technology, Karolinska Institutet, Stockholm, Sweden; 18 CMIV, Centre of Medical Image Science and Visualization, Linköping University, Linköping, Sweden; 19 Department of Clinical Physiology; Department of Health, Medicine and Caring Sciences, Linköping University, Linköping, Sweden; 20 Department of Molecular Medicine and Surgery, Karolinska Institutet, Stockholm, Sweden; 21 Division of Cardiovascular Medicine Unit, Department of Medicine Solna, Karolinska Institute, Stockholm, Sweden; 22 Department of Clinical Science, Danderyd University Hospital, Stockholm, Sweden; 23 Department of Clinical Genetics and Genomics, Region Västra Götaland, Sahlgrenska University Hospital, Gothenburg, Sweden; 24 Department of Medical Sciences, Cardiology, Uppsala University, Uppsala, Sweden; 25 Uppsala Clinical Research Center, Uppsala University, Uppsala, Sweden; 26 Department of Cardiology, Skåne University Hospital, Malmö, Sweden; 27 Pulmonary Department, Sleep Disorders Center, Sahlgrenska University Hospital, Gothenburg, Sweden; 28 Center of Sleep and Wake Disorders, Sahlgrenska Academy, Gothenburg University, Göteborg, Sweden; 29 Department of Cardiology, Department of Health, Medicine and Caring Sciences, Unit of Cardiovascular Sciences, Linköping University, Linköping, Sweden; 30 Department of Clinical Sciences, Danderyd University Hospital, Karolinska Institutet, Stockholm, Sweden; 31 Department of Medical Sciences, Clinical Epidemiology, Uppsala University, Uppsala, Sweden; 32 Department of Medical Sciences, Respiratory, Allergy and Sleep Research, Uppsala University, Uppsala, Sweden; 33 Heart and Vascular Theme, Department of Cardiology, and Clinical Genetics, Karolinska University Hospital, Stockholm, Sweden; 34 Department of Radiology, Department of Health, Medicine and Caring Sciences, Linköping University, Linköping, Sweden; 35 Department of Clinical Sciences, Huddinge University Hospital, Karolinska Institute, Stockholm, Sweden; 36 Respiratory Medicine, Department of Medical and Health Sciences (IMH), Linköping University, Linköping, Sweden; 37 Department of Internal Medicine, Skåne University Hospital, Malmö, Sweden; 38 Department of Community Medicine and Rehabilitation, Geriatric Medicine, Umeå University, Umeå, Sweden; 39 Department of Medicine Geriatrics and Emergency Medicine, Sahlgrenska University Hospital Östra Hospital, Gothenburg, Sweden; 40 Department of Public Health and Clinical Medicine, Umeå University, Umeå, Sweden; 41 Department of Respiratory Medicine and Allergy, Karolinska University Hospital Solna, Stockholm, Sweden; 42 Respiratory Medicine Unit, Department of Medicine Solna and Center for Molecular Medicine, Karolinska Institutet, Stockholm, Sweden; 43 Section of Occupational and Environmental Medicine, School of Public Health and Community Medicine, Institute of Medicine, Sahlgrenska Academy, University of Gothenburg, Gothenburg, Sweden; 44 Department of Occupational and Environmental Medicine, Sahlgrenska University Hospital, Gothenburg, Sweden; 45 Department of Translational Medicine, Lund University, Malmö, Sweden; 46 Department of Health, Medicine and Caring Sciences, Linköping University, Linköping, Sweden; 47 The George Institute for Global Health, University of New South Wales, Sydney, Australia

**Keywords:** Atherosclerosis, Spirometry, Emphysema, Coronary heart disease

## Abstract

Reduced lung function is associated with cardiovascular mortality, but the relationships with atherosclerosis are unclear. The population-based Swedish CArdioPulmonary BioImage study measured lung function, emphysema, coronary CT angiography, coronary calcium, carotid plaques and ankle-brachial index in 29,593 men and women aged 50–64 years. The results were confirmed using 2-sample Mendelian randomization. Lower lung function and emphysema were associated with more atherosclerosis, but these relationships were attenuated after adjustment for cardiovascular risk factors. Lung function was not associated with coronary atherosclerosis in 14,524 never-smokers. No potentially causal effect of lung function on atherosclerosis, or vice versa, was found in the 2-sample Mendelian randomization analysis. Here we show that reduced lung function and atherosclerosis are correlated in the population, but probably not causally related. Assessing lung function in addition to conventional cardiovascular risk factors to gauge risk of subclinical atherosclerosis is probably not meaningful, but low lung function found by chance should alert for atherosclerosis.

**Supplementary Information:**

The online version contains supplementary material available at 10.1007/s10654-023-01088-z.

## Introduction

Reduced lung function is a risk factor for cardiovascular mortality and morbidity [1–5]. The increased mortality risk has been observed also for moderately reduced lung function measures, well within the normal range, and results have usually persisted after adjustment for smoking and other cardiovascular risk factors [1–5]. This could have important implications for cardiovascular risk assessment and prevention, as lung function may reveal important information about an individual’s cardiovascular status, and spirometry has been proposed to be used in routine health screening to identify individuals with high cardiovascular risk [1–4]. If associations were causal, novel cardiovascular risk assessment tools [2] or pharmaceutical treatment regimens may be investigated [6, 7].

Associations of subclinical lung function impairments with atherosclerosis may shed light on disease burden and prevention windows. The only population-based study hitherto examining associations of lung function with atherosclerosis in carotid, coronary and peripheral arteries [8] observed that lower forced expiratory volume in 1 s (FEV_1_) and emphysema were associated with carotid and peripheral artery atherosclerosis, but not with coronary artery calcification [8]. Many vulnerable coronary plaques are not calcified, hence coronary calcification measures do not give full information about the extent and severity of coronary atherosclerosis.

Different aspects of lung impairment may differentially predict atherosclerotic disease [9]. Very few population-based studies have examined whether reduced diffusing capacity is associated with subclinical atherosclerosis. A study of 450 smokers and non-smokers from the general population reported increased occurrence of carotid plaques in individuals with low CO diffusing capacity, but no significant relationship between carotid plaque and FEV_1_ or chronic obstructive pulmonary disease (COPD) status [10]. In contrast, a study of endothelial dysfunction, a putative precursor of atherosclerosis, found no significant relationship with CO diffusing capacity [11].

Mendelian randomization (MR) is a method that uses genetic variants as causal anchors to obtain estimates for observational associations in the presence of potential confounding [12]. Previous MR studies of lung function have reported significant inverse relationships between genetic risk scores for lung function and blood pressure and diabetes, i.e., two important risk factors for atherosclerosis [13, 14], and with acute coronary events [15].

We aimed to examine the associations of emphysema and results from pulmonary function tests with atherosclerosis in multiple arterial beds in a contemporary population-based cohort with a large subsample of never-smokers. In addition, we examined potential causal relationships using bi-directional two-sample Mendelian randomization (MR) analyses.

## Methods

### Cohort study sample

The Swedish CArdioPulmonary bioImage Study (SCAPIS) is a collaboration between six Swedish universities and university hospitals, with the aim of studying cardio-pulmonary diseases in a large population-based cohort. Randomly selected individuals from the general population, who were between 50 and 64 years old and living in six urban areas surrounding the university hospitals, received an invitation letter [16]. The study subjects should be able to understand instructions and complete questionnaires, as judged by the study staff, but no other exclusion criteria were applied. Overall participation rate was approximately 50% and a total of 30,154 men and women were included in the study. Subjects were examined at one of the screening centres during 2013–2018. The study was approved as a multi-center study by the ethical review board in Umeå (Dnr 2010-228-31 M). All participants gave written informed consent.

After exclusion of individuals with no CT examination or who did not perform pulmonary function tests, 29,593 individuals, 15,175 women and 14,418 men, remained for this study. Since the distribution of lung function measures and prevalence of atherosclerosis differ markedly by sex, all analyses were done separately in men and women. Given the fact that smoking is a common cause of reduced lung function as well as atherosclerosis, we also repeated all analyses in never smokers.

### Clinical investigations

Information on smoking and medical treatment for hypertension or hyperlipidaemia were derived from the questionnaire. Smoking status was categorized as current smoker, former smoker and never smoker, respectively. Physical activity was measured over seven consecutive days using ActiGraph GT3X or GT3X + activity monitors [17]. The proportion of sedentary time was calculated for each individual and used as a covariate in this study. Subjects were classified as having diabetes based on responses in questionnaire and blood tests for HbA1c and p-glucose. Subjects with previously known diabetes, elevated p-glucose (≥ 7.0 mmol/L) or HbA1c (≥ 48 mmol/mol) were classified as having diabetes [18, 19].

Body weight was measured on digital scales with subjects dressed in light indoor clothing without shoes. Body height was measured to the nearest centimetre using a stadiometer. Waist circumference was measured midway between the palpated iliac crests and the palpated lowest rib margins. Body mass index (BMI) was calculated as body weight/height^2^ (kg/m^2^). Systolic and diastolic blood pressure (SBP, DBP) was measured in the supine position twice in each arm with an Omron M10-IT automatic device (www.omron.com), with one minute between the two measurements. Mean SBP and DBP from the arm with the highest mean SBP was used in the analysis. A fasting venous blood sample was collected for analysis of lipids and C-reactive protein (CRP). The analyses were performed using standard methods at the laboratory of the university hospital. All laboratories were accredited either according to ISO/IEC 17025 or to 15189 by the Swedish Board for Accreditation and Conformity.

### Lung function assessments

Dynamic spirometry (Jaeger MasterScreen PFT, Carefusion, Hoechberg, Germany) was performed 15 min after bronchodilation using 400 μg of Salbutamol with subjects in the sitting position and wearing a nose clip. Forced expiratory volume in 1 s (FEV_1_), forced vital capacity (FVC), and ratio of FEV_1_/FVC were measured according to the American Thoracic Society (ATS) and European Respiratory Society (ERS) standards [20].

Carbon monoxide (CO) uptake was measured using the single-breath CO diffusing capacity (D_LCO_) test (Jaeger MasterScreen PFT).

Computed tomography (CT) imaging in SCAPIS has been described in detail previously [16]. CT was performed using a dual-source CT scanner equipped with a Stellar Detector (Somatom Definition Flash, Siemens, Forchheim, Germany). The software and hardware of the CT scanners was identical at the six sites. Visual scoring of emphysema from the CT images was done by radiologists blinded to all clinical data, according to a modified score sheet used in the COPD Gene study (www.copdgene.org). Each lung was divided into three regions and each of the 6 regions were assessed for emphysema that was graded as 1 (mild emphysema), 2 (moderate emphysema) or 3 (severe emphysema) [21]. Absence of emphysema was given 0 points. The points were summed for each individual with a maximum emphysema score of 18.

### Atherosclerosis assessments

Trained technicians scanned both carotid arteries for plaque in the common carotid artery, the internal carotid artery, the external carotid artery, using Siemens Acuson S2000. Carotid plaque was defined as a focal structure protruding into the arterial lumen of at least 0.5 mm or 50% of the surrounding intima media thickness (IMT) value, or demonstrating a thickness > 1.5 mm as measured between the intima-lumen interface and media-adventitia interface [22]. Any carotid plaque (vs. no plaque) was used as a binary variable in the analysis.

Ankle-brachial blood pressure index (ABI) was measured bilaterally using a Doppler pulse sensor (Hadeco Bidop ES-100V3, www.hadeco.co.jp) with the subject in the supine position. SBP was measured manually and twice bilaterally in the dorsalis pedis and posterior tibial arteries. Ankle brachial index (ABI) was calculated as the ratio between ankle and brachial systolic pressures for the arm with the highest mean SBP. The leg with the lowest ABI was used in the analysis.

Cardiac CT imaging was performed using electrocardiogram-gated non-contrast CT imaging at 120 kV as well as contrast enhanced coronary CT angiography (CCTA).

All non-contrast image sets were reconstructed and coronary calcium was identified and scored using the syngo.via calcium scoring software (Siemens, Erlangen, Germany) [16]. The area of calcification of each 3 mm slice was multiplied with an intensity factor and summed up to a coronary artery calcium score (CACS) for the artery tree according to Agatston [23].

For CCTA, a β-blocker (metoprolol) and sublingual glyceryl nitrate were given for control of heart rate and dilation of coronary arteries. The contrast medium iohexol (GE Healthcare, 350 mg I/mL) was given at a dose of 325 mg I/kg body weight. All contrast-enhanced CCTA image sets were reconstructed (B35f HeartView medium CaScore) and visually scored for atherosclerosis.

The cardiac CT images were evaluated by trained thoracic radiologists or cardiologists with between 1 and > 10 years of training in reading CCTA and a competence level of 1–3 according to the American College of Cardiology Foundation/American Heart Association Clinical Competence Statement on cardiac CT [16]. For reporting of coronary plaque burden from CCTA, we used the 18 coronary segment model defined by the Society of Cardiovascular Computed Tomography. To increase quality of the most important findings, readers focused on the 11 clinically most relevant segments (segments 1–3, 5–7, 9, 11–13, 17), which were compulsory to report. Other segments were only reported if they had atherosclerosis or calcium blooming. The coronary segments were visually examined for presence of plaques and classified as: no atherosclerosis; 1–49% stenosis; ≥ 50% (i.e. significant) stenosis; not assessable because of calcium blooming, not assessable due to technical failure or segment missing. Luminal obstruction was defined visually by estimating the longest and shortest diameter at the site of stenosis. Segment involvement score (SIS) was calculated as the number of coronary segments with plaque (i.e., vessels with 1–49% or ≥ 50% stenosis or vessels not assessable because of calcium blooming) [24].

### Statistical analysis

Descriptive statistics are presented as medians with interquartile ranges for continuous measures and numbers (%) for categories. Associations between the exposures and the outcomes were assessed using the cumulative probability model described by Liu et al. [25] using a logit link function. The model is an ordinal logistic model, which directly models the cumulative distribution function and is invariant of any monotone transformation of the outcome. The model fit will thus be the same if the outcome is transformed in a way such that the orderings of the outcome is preserved, e.g., log or square root transformation. This allows conditional mean values, quantiles and exceedance probabilities for any cutoff to be calculated from a single model fit.

**Fig. 1:**
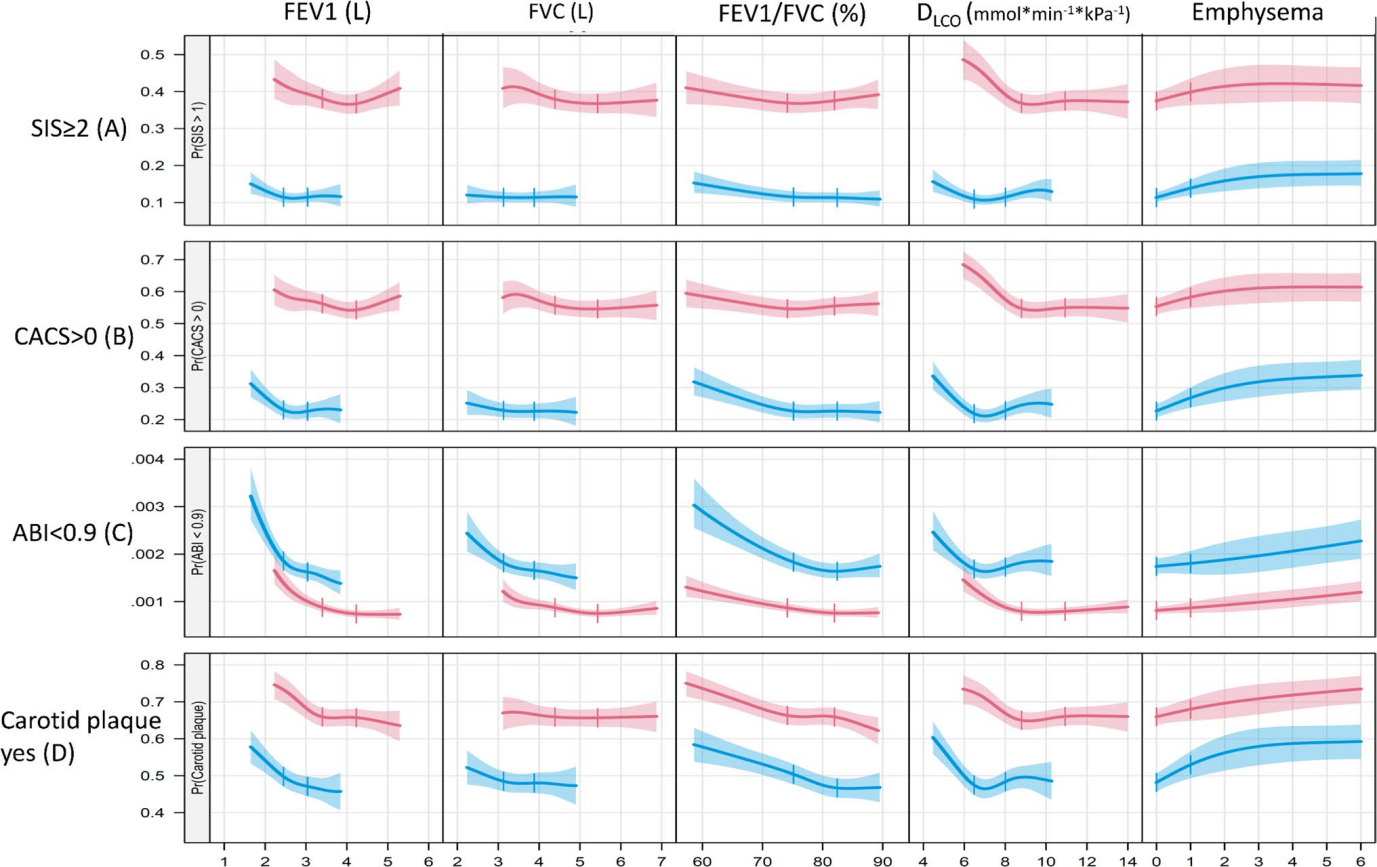
Associations of lung function and emphysema score with atherosclerosis in three arterial beds. Associations between exposures (columns) and outcomes (rows) in men (red) and women (blue). Depicted are the probability of SIS >  = 2 (top row, **A**), probability of CACS > 0 (**B**), probability of ABI < 0.9 (**C**) and the probability of plaque in at least one coronary artery (**D**). Curves are truncated at the sex-specific 10th and 90th percentiles of the exposure variables and vertical broken lines indicate the sex specific interquartile range (IQR) with the exception of the Emphysema score where the IQR equals zero and lines are drawn at zero and one

One model was fit for each combination of exposure and outcome, adjusting for site, age, weight, height, waist circumference, CRP, smoking status (never, former, current), diabetes mellitus, fraction of the day spent sedentary and education level. A directed acyclic graph was used to identify potential confounding factors and minimize bias of the multivariate model (Online resources Fig. 1). Exposure variables from the pulmonary function tests were modeled using restricted cubic splines.

As the distributions differed between men and women for FEV_1_, FVC and D_LCO_, the knots were placed at the 5th and 35th percentiles of each variable’s distribution in women; the overall median and the 65th and 95th percentiles of each variable’s distribution in men. The knots for FEV_1_/FVC were placed at the 5th, 35th, 65th and 95th percentiles of the marginal distribution and the knots for emphysema score were placed at scores 0, 1 and 5.

All other continuous variables were modeled using restricted cubic splines with knots placed at the 10th, 50th and 90th percentiles. Sex specific estimates were obtained by including a multiplicative interaction with sex in all models. The odds ratio (OR; 95% confidence intervals) of higher CACS, lower ABI, carotid plaques and higher SIS are expressed in relation to a sex-specific interquartile range (IQR) increment of FEV_1_, FVC, FEV_1_/FVC and D_LCO_. For emphysema score ORs are shown for an increase from zero to one. The figures show the estimated probabilities of CACS > 0, ABI < 0.9, plaque in at least one carotid artery and SIS >  = 2.

Missing values were imputed under fully conditional specification and coefficients and standard errors were calculated using Rubin’s rules. The ratio FEV_1_/FVC was imputed using passive imputation, i.e., the ratio was calculated for each imputed data set after both FEV_1_ and FVC had been imputed.

### Mendelian randomization study

Mendelian randomization (MR) is a method that uses genetic variants as causal anchors to obtain estimates for observational associations in the presence of potential confounding [12]. This technique was used to examine potentially causal effects of lung function on atherosclerosis, and vice versa, using data from the UK Biobank and the Cohorts for Heart and Aging Research in Genomic Epidemiology and Million Veteran Program consortia (see Online resource methods). In the primary MR analysis, genetic variants associated with FEV_1_, FVC and FEV_1_/FVC were used as genetic instruments to assess the causal effect of lung function on atherosclerosis (i.e., carotid Intima-Media Thickness (IMT), presence of carotid plaques, and peripheral arterial disease (PAD), using two-sample random effects inverse-variance weighted MR. Next, genetic variants associated with carotid IMT and PAD were used as genetic instruments to assess the causal effect of atherosclerotic disease on lung function (FEV_1_, FVC, FEV_1_/FVC).

Additionally, we used random effects MR-Egger, weighted-median MR, and CAUSE MR to assess pleiotropic effects; multivariable MR to specifically assess the potential effect of smoking and height. We repeated the random effects inverse-variance weighted MR analysis in never-smokers, using genetic instruments from never-smokers of the UK Biobank. Details of the MR analysis are presented in the Online resources.

## Results

**Fig. 2:**
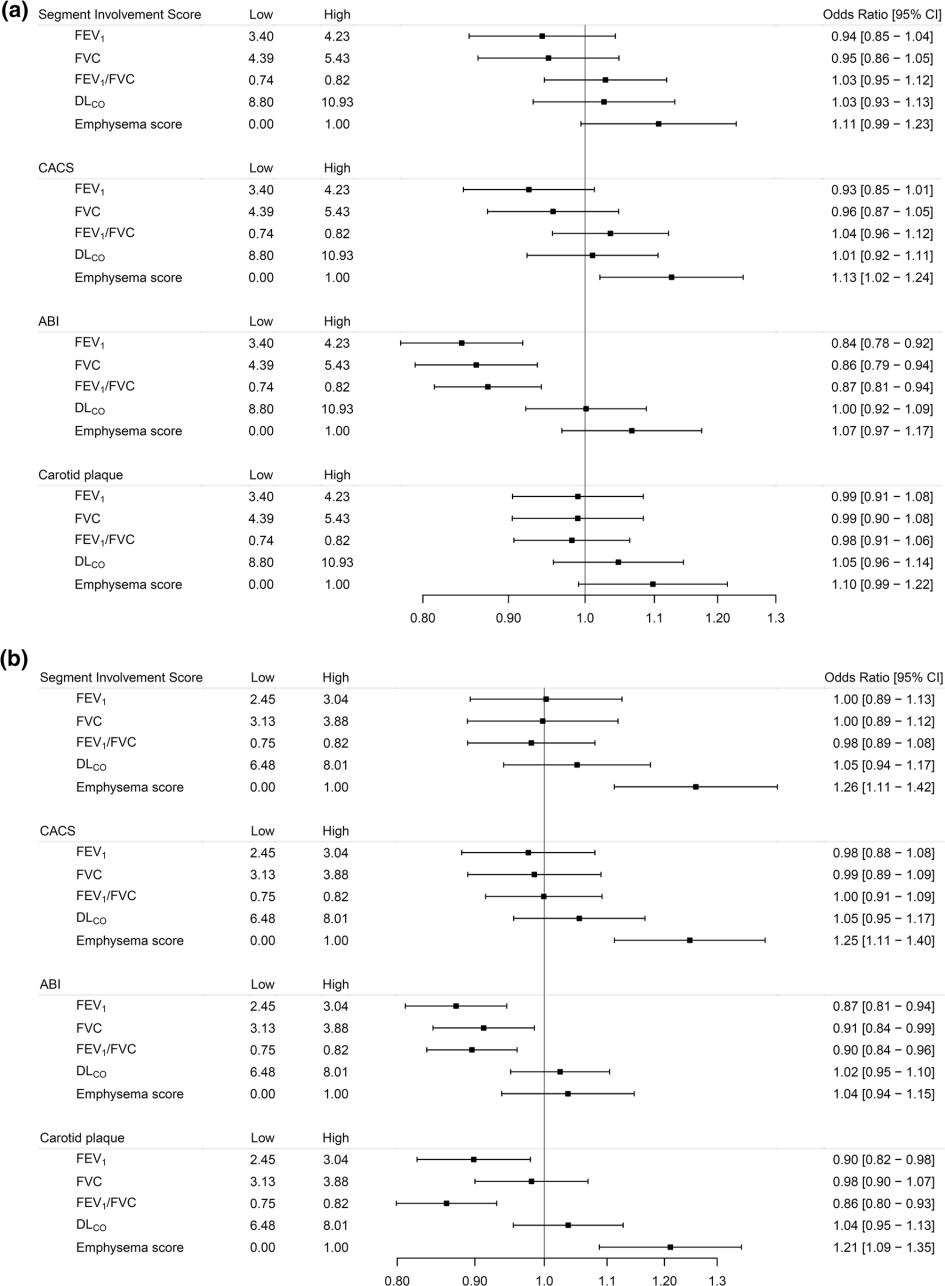
Forest plots of all men (**a**) and women (**b**). Adjusted OR (95% CIs) for a sex-specific interquartile range increase in the exposures with the exception of the Emphysema score where the OR corresponds to an increase between zero and one. ORs are adjusted for site, age, weight, height, waist circumference, CRP, smoking status (never, former, current), diabetes mellitus, fraction of the day spent sedentary and education. "Low" and "high" indicate the interquartile range for the exposure variables

A total of 29,593 individuals, 15,175 women and 14,418 men, were included in the study. A total of 3648 (12.3%) were current smokers, 10,445 (35.3%) were former smokers, 14,524 (49.1%) were never smokers and smoking status was unknown for 976 (3.3%) participants. The characteristics of the study population are presented in Table 1. Cardiovascular disease risk factors were generally more prevalent in individuals with lower lung function (Online resources Tables 1–4). Substantial differences were observed for SBP and DBP, diabetes, CRP and smoking across quintiles of FEV_1_. The distribution of atherosclerosis in three vascular beds in relation to a commonly used cut-off for COPD (FEV_1_/FVC < 0.7) and positive emphysema score is illustrated in Online resources Fig. 2.

**Table 1:** Characteristics of the study cohort

	All	Female	Male
n	29,593	15,175	14,418
Age (years)	57.4 [53.7; 61.2]	57.4 [53.7; 61.2]	57.5 [53.7; 61.3]
Height (cm)	172 [165; 179]	166 [161; 170]	179 [175; 184]
Weight (kg)	79.0 [68.8; 90.0]	70.4 [63.0; 80.0]	86.8 [79.0; 96.0]
BMI (kg/m^2^)	26.4 [23.9; 29.4]	25.6 [23.0; 29.1]	26.9 [24.8; 29.6]
Smoking status (%)			
Current smoker	3648 (12.3)	1880 (12.4)	1768 (12.3)
Former smoker	10,445 (35.3)	5752 (37.9)	4693 (32.5)
Never smoker	14,524 (49.1)	7091 (46.7)	7433 (51.6)
Unknown	976 (3.3)	452 (3.0)	524 (3.6)
College or University degree (%)	13,017 (45.1)	7316 (49.2)	5701 (40.7)
Systolic BP (mmHg)	124 [114; 136]	121 [110; 134]	127 [118; 138]
Diastolic BP (mmHg)	77 [70; 84]	76 [69; 84]	78 [72; 85]
Anti-hypertensive medication (%)	5688 (19.9)	2693 (18.3)	2995 (21.6)
LDL cholesterol (mmol/L)	3.4 [2.8; 4.0]	3.4 [2.8; 4.0]	3.4 [2.8; 4.0]
HDL cholesterol (mmol/L)	1.6 [1.3; 1.9]	1.8 [1.5; 2.1]	1.4 [1.1; 1.6]
Lipid-lowering medication (%)	2268 (7.9)	893 (6.1)	1375 (9.9)
Diabetes (%)	2228 (7.5)	834 (5.5)	1394 (9.7)
CRP (mg/L)	1.0 [0.60; 2.2]	1.0 [0.60; 2.3]	1.0 [0.60; 2.0]
Sedentary time (% of day)	55.0 [47.0; 61.0]	53.0 [45.0; 59.0]	57.0 [49.0; 63.0]
FEV_1_ (liter)	3.18 [2.69; 3.81]	2.74 [2.45; 3.04]	3.81 [3.40; 4.23]
FVC (liter)	4.08 [3.44; 4.91]	3.49 [3.13; 3.88]	4.91 [4.39; 5.43]
FEV_1_/FVC	0.79 [0.75; 0.82]	0.79 [0.75; 0.82]	0.78 [0.74; 0.82]
D_LCO_ (mmol/(min kPa))	8.32 [7.06; 9.88]	7.22 [6.48; 8.01]	9.86 [8.80; 10.93]
ABI	1.21 [1.15; 1.27]	1.19 [1.13; 1.25]	1.23 [1.17; 1.29]
ABI < 0.9 (%)	77 (0.3)	28 (0.2)	49 (0.3)
Segment Involvement Score ≥ 2 (%)	3173 (14.8)	789 (7.1)	2384 (22.9)
Carotid plaque n (%)	16,131 (55)	7361 (49)	8770 (61)
CACS 0 n (%)	14,957 (59.8)	9207 (72.7)	5750 (46.6)
CACS 1–99 n (%)	7140 (28.5)	2754 (21.7)	4386 (35.5)
CACS ≥ 100 n (%)	2916 (11.7)	705 (5.6)	2211 (17.9)

BMI, body mass index; BP blood pressure; LDL low density lipoprotein cholesterol; HDL, high density lipoprotein cholesterol; CRP C-reactive protein; FEV1 Forced expiratory volume (1 s), FVC forced vital capacity; ABI ankle brachial blood pressure index; CACS coronary artery calcium score. Values are n (%) or median [interquartile range]

### Associations of lung function with coronary atherosclerosis

**Fig. 3:**
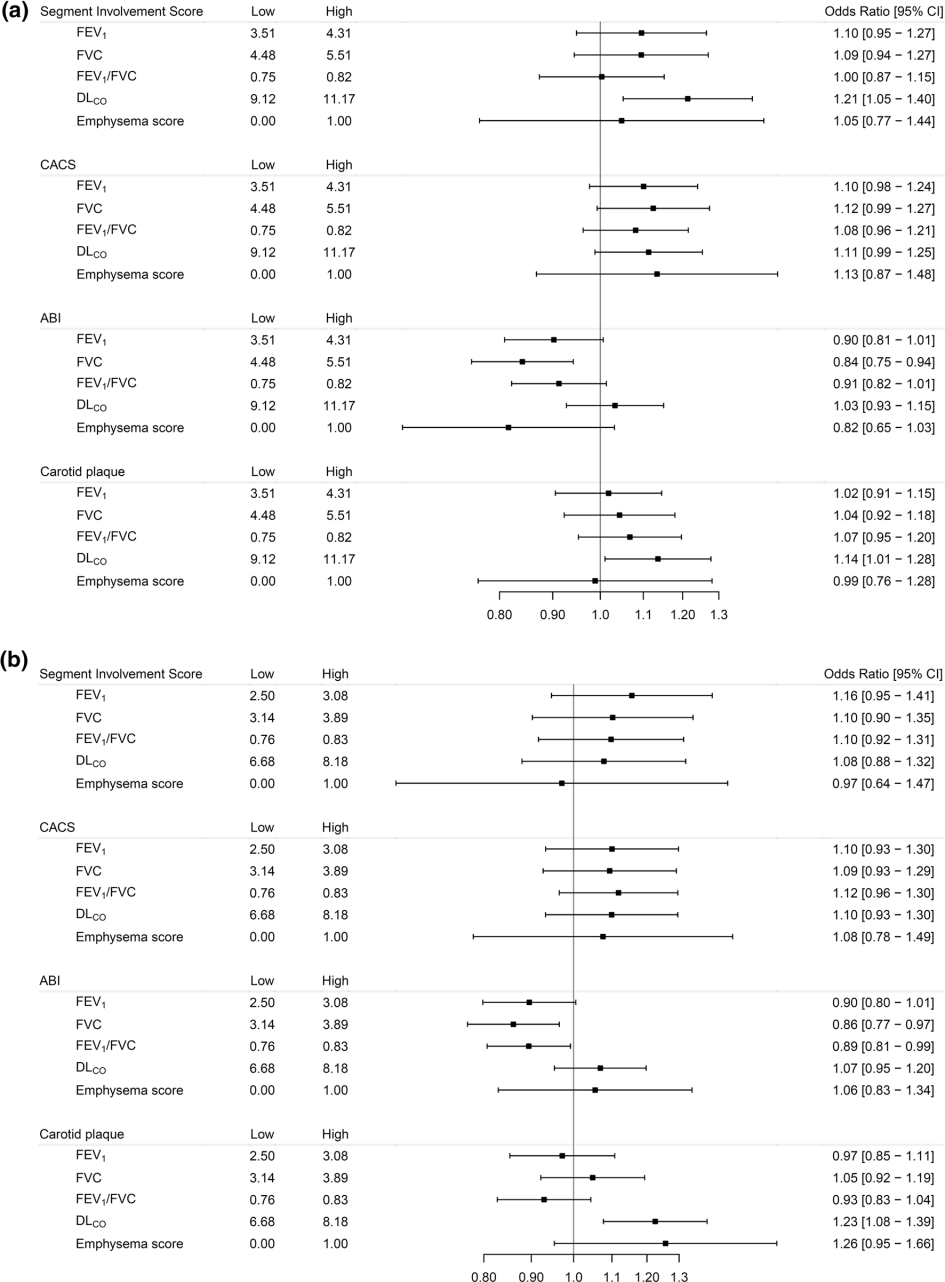
Forest plots of men (**a**) and women (**b**) who never smoked. Adjusted OR (95%CIs) for a sex-specific interquartile range increase in the exposures with the exception of the Emphysema score where the OR corresponds to an increase between zero and one. ORs are adjusted for site, age, weight, height, waist circumference, CRP, diabetes mellitus, fraction of the day spent sedentary and education. "Low" and "high" indicate the interquartile range for the exposure variables

The associations of measures of lung function with coronary atherosclerosis, adjusted for age and height, are presented for men and women in Online resources Fig. 3, and after full adjustments for risk factors in Figs. 1 and 2a, b. Both in men and women, lower FEV_1_, FVC and positive emphysema score were associated with coronary atherosclerosis adjusted for age and height (Online resources Fig. 3). Adjusting also for cardiovascular disease risk factors, positive emphysema score was associated with CACS (in men and women) and SIS (in women) (Fig. 2a, b), while no significant association remained for FEV_1_ and FVC.

**Fig. 4:**
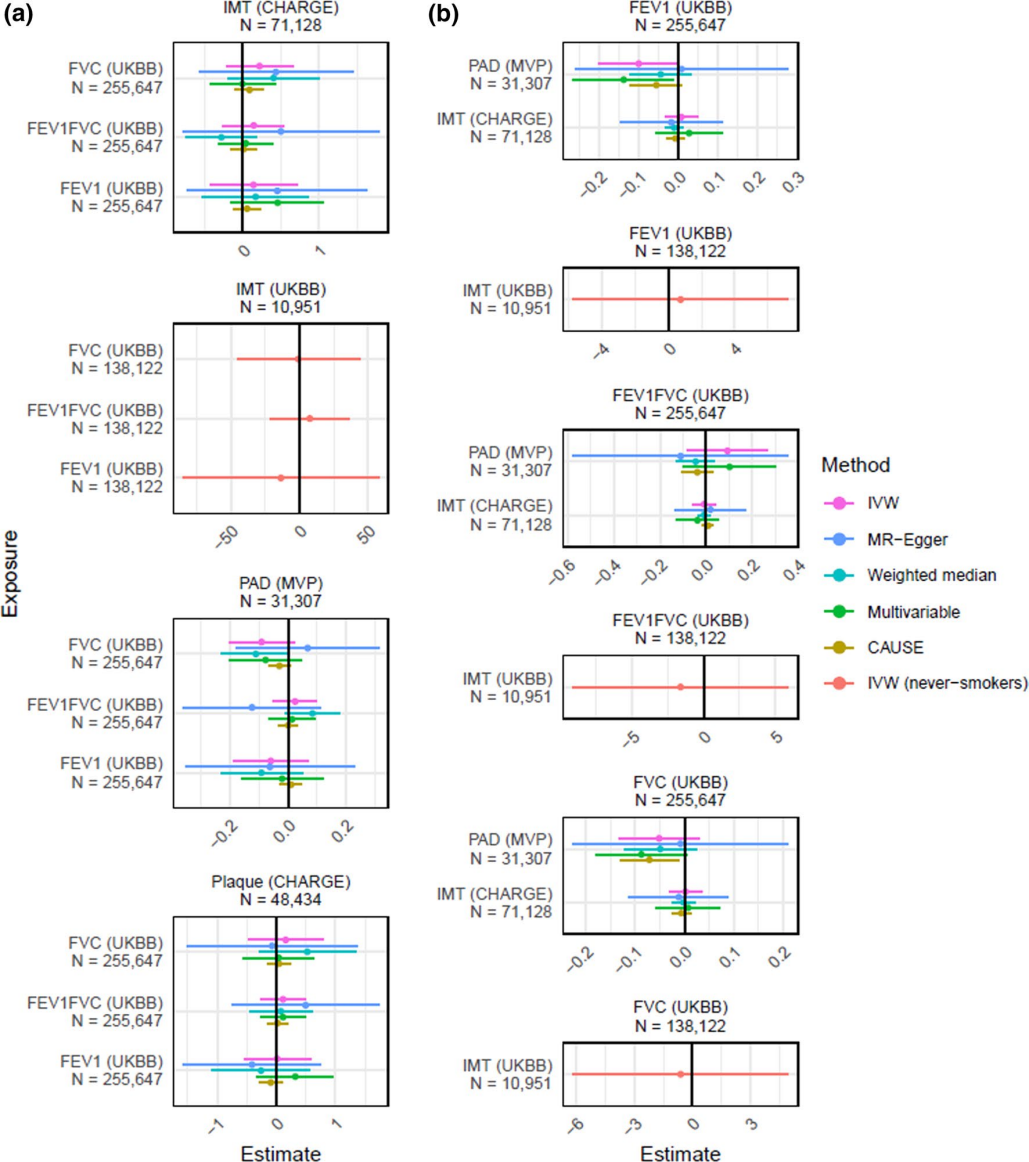
Causal effects and 95% confidence intervals for (**A**) the effect of lung function on atherosclerosis and (**B**) the effect of atherosclerosis on lung function, estimated using Mendelian randomization (IVW, MR-Egger, Weighted median MR, CAUSE MR, Multivariable MR, and IVW in never-smokers). Estimates above 0 indicate a positive effect, and below 0 a negative effect. Please see Online resource for details

We did not observe any associations of emphysema score or lower lung function with coronary atherosclerosis in the subgroup of 14,524 never-smokers (Fig. 3a, b). A significant positive relationship between D_LCO_ and SIS was observed in never smoking men. However, the relationships between D_LCO_ in never smoking men and SIS and CACS, respectively, was non-linear with a nadir around the 25th percentile, and very low D_LCO_ was associated with high SIS and CACS scores (Online resources Fig. 4).

### Associations of lung function with carotid plaques

Adjusting for cardiovascular disease risk factors, lower FEV_1_, lower FEV_1_/FVC and positive emphysema score were associated with presence of carotid plaques in women. No significant relationship was observed in men after adjustments for cardiovascular risk factors (Fig. 2a, b). These relationships were non-significant in never smokers. However, a significant positive relationship between D_LCO_ and presence of carotid plaques was observed in the analysis restricted to never smokers, both in men and women (Fig. 3a, b, Online resources Fig. 4).

### Associations of lung function with ankle-brachial index

Lower FEV_1_, FVC and FEV_1_/FVC were all associated with increased odds of having abnormal ABI (ABI < 0.9) in both men and women (Figs. 1 and 2a, b), after adjustments for potential confounders. Positive emphysema score was associated with increased odds of ABI < 0.9 after adjustments for age and height (Online resources Fig. 3a, b), but not after adjustment for cardiovascular risk factors (Fig. 2a, b).

In never-smokers, only lower FEV_1_/FVC and FVC in women, and lower FVC in men, were significantly associated with ABI < 0.9 (Fig. 3a, b).

### Mendelian randomization study

Genetic variants associated with FEV_1_, FVC and FEV_1_/FVC were used as instruments to assess the causal effect of lung function on atherosclerosis (carotid IMT, presence of carotid plaques, PAD) using two-sample random effects inverse-variance weighted MR (Online resources Table 5). We found no evidence for an effect of lung function on atherosclerosis (Fig. 4). Genetic variants associated with carotid IMT and PAD were used as genetic instruments to assess the causal effect of atherosclerosis on lung function (FEV_1_, FVC, FEV_1_/FVC; Online resources Table 5). Similarly, we found no evidence for an effect of atherosclerosis on lung function. The results were essentially unchanged in analysis of never smokers and after assessments of potential pleiotropic effects using MR-Egger, weighted-median MR, and CAUSE MR. We found no evidence for an effect of lung function on atherosclerosis or vice versa (Fig. 4).

## Discussion

### Principal observations

In this well-characterized population-based study with a large never-smoking subsample, we found that lower FEV_1_ and FVC were associated with increased odds of ABI < 0.9. Lower FEV_1_ was also associated with presence of carotid plaque in women. However, except for emphysema score, the relationships with coronary atherosclerosis were attenuated after adjustments for other cardiovascular risk factors. Furthermore, coronary atherosclerosis was not associated with lower lung function or emphysema in a subgroup of 14,524 never smokers. In line with the results from our observational study, we found no evidence for a causal effect of lung function on atherosclerosis, or vice versa, in our Mendelian Randomization study. Overall, the relationships that we found between measures of lung function and subclinical atherosclerosis were to a major extent explained by other risk factors for atherosclerosis.

### Lung function, emphysema and subclinical coronary disease

Our results for lung function and coronary atherosclerosis are largely consistent with results from the MESA study [8], which examined spirometry results and percentage emphysema in relation to CACS. No differences in CACS between those with and without airflow obstruction were observed in two other studies [26, 27]. In contrast, low lung function was associated with CACS in a cohort of Korean men undergoing annual health check-ups [28]. It should be noted that the degree of control for cardiovascular risk factors have varied between studies and few have specifically addressed a large group of never smokers [8]. D_LCO_ was not associated with coronary atherosclerosis in the full sample. The present study extend the results from previous studies by including non-calcified coronary plaque in the analysis, by including measures of diffusing capacity, and by analyzing a very large subsample of never smokers.

### Lung function, carotid plaque and subclinical peripheral arterial disease

Previous studies have indicated that the relationship between lung function and atherosclerosis could be stronger in the carotid or peripheral circulation than in coronary vessels [8, 26, 27]. However, no association between spirometry findings and carotid plaques was reported from the Atherosclerosis Risk in Communities study [29]. In our study, prevalence of carotid plaques was associated with FEV_1_ and FEV_1_/FVC in women, but no significant associations were found in men. Low lung function was not associated with carotid plaques in never-smokers.

Low diffusing capacity of the lungs has previously been associated with presence of carotid plaque [10] and with increased prevalence of cardiovascular disease in respiratory outpatients [30], but no significant relationship was found for diffusing capacity in a study of endothelial function [11]. A study of 413 current and former smokers reported a non-significant relationship between D_LCO_ and coronary calcium after risk factor adjustment [31]. Both for carotid plaque and coronary atherosclerosis, we found non-linear relationships with D_LCO_ in the subgroup of never smokers, with a nadir around the 25th percentile. We do not have any obvious explanation for this non-linearity. However, it could be speculated that there is a threshold effect for the relationship between D_LCO_ and atherosclerosis, and that most individuals in a never-smoking population have D_LCO_ above this threshold.

Lower FEV_1_, FVC and FEV_1_/FVC were associated with ABI, and for FVC, this persisted in analysis of never smokers. ABI was similarly associated with FEV_1_ and FVC in studies by Barr et al. [8] and Schroeder et al. [29].

### Lung function and clinical coronary disease events

The weak or absent relationship between lung function and coronary atherosclerosis is seemingly in contradiction to the increased incidence and mortality due to ischemic heart disease, which has been reported in many previous studies [1–5]. A recent Mendelian randomization study using data from the Coronary Artery Disease Genome wide Replication and Meta-analysis plus The Coronary Artery Disease Genetics (CARDIoGRAMplusC4D) suggested an inverse relation between lung function, measured by FEV_1_ or FVC, and coronary artery disease, defined as patients with a diagnosis of myocardial infarction, acute coronary syndrome, chronic stable angina, or coronary stenosis > 50% [13]. However, the relationship with FEV_1_ was attenuated and non-significant after adjustment for height. Another recent Mendelian randomization study, using genetic data from UK Biobank, reported a significant relationship between FVC (but not FEV_1_) and coronary heart disease [15]. Previous studies have also shown inverse relationships between FEV_1_ and incidence of hypertension and diabetes [32, 33], in line with the cross-sectional associations found in this study. This was recently supported by two Mendelian randomization studies, which reported significant inverse relationships between genetic risk scores for lung function and blood pressure and diabetes, respectively [13, 14]. This supports the view that other cardiovascular risk factors partly explain the relationships between low lung function and cardiovascular events.

The pathophysiology of a clinical coronary event is complex and involves both development of atherosclerosis and plaque rupture; atherosclerosis is usually asymptomatic whereas plaque rupture is the usual mechanism in an acute coronary event. Low lung function could hypothetically be related to plaque rupture or plaque erosion rather than coronary atherosclerosis. Furthermore, it is possible that other risk factors, such as ventricular arrhythmia are responsible for the increased cardiac mortality rates in subjects with low lung function. Population-based studies from “Men born in 1914” [34] and the Cardiovascular Health Study [35] have reported significant associations between FEV_1_ or FVC and ventricular arrhythmias. In the study “Men born in 1914”, both the occurrence and prognostic significance of cardiac arrhythmias were associated with lung function. It also is noteworthy that the relationship between low lung function and incidence of coronary events has been shown to be stronger for sudden cardiac deaths than for non-fatal events [36], which supports the hypothesis that ventricular arrhythmia could play a role. Hence, low lung function could be linked to cardiac mortality through other pathways than atherosclerosis. Although the overall results of the present study do not support a causal role of lung function for development of atherosclerosis, poor lung volumes in a dynamic spirometry could still be reason for a global evaluation of atherosclerotic risk factors to reduce the cardiovascular risk.

### Strengths and limitations

The MR analyses largely confirmed the results from the observational analyses; there was no evidence of a causal effect of lung function on atherosclerosis or vice versa. The MR technique can be very useful for studying relationships between exposure and outcome in an unbiased study design. However, there are also important limitations. A limitation of the present study is that relatively few genetic polymorphisms were available for measures of atherosclerosis. The genetic polymorphisms were taken from genome wide association studies with different methods for assessment of atherosclerosis and no valid genetic instrument was available for CACS or SIS. Also, the genetic instruments for lung function and atherosclerosis explain a relatively low proportion of their variance, which makes the analysis potentially underpowered. The genetic analysis among never-smokers is limited by a relatively small sample size and the exposure and outcome variables are from the same study (UK Biobank), making the estimates potentially inaccurate or biased. Furthermore, even in subjects without apparent cardiovascular disease, subclinical reductions in cardiac function could cause reduced lung volumes [37]. This type of reverse causation could potentially bias correlations between genetic instruments of lung volumes and cardiac disease and cannot be completely excluded in studies with two-sample MR design [38].

The size of the study and the detailed characterization of subclinical atherosclerosis and lung function are important strengths of the study. The cohort included large numbers of never-smokers, which is another major strength in a study of lung function. The spirometries were performed post-bronchodilation according to guidelines. Reduced lung volumes in this study can therefore be assumed to reflect chronic rather than reversible conditions. Detailed information about cardiac arrhythmia was not available, which is a limitation. Such information could further clarify the relationship between lung function and acute coronary events.

The men and women in the study population were 50–64 years old, i.e., age group in which subclinical atherosclerosis is prevalent, and for which preventive measures still could change the course of the disease. However, it is unclear whether relationships are similar in older age-groups, when incidence of clinical events is higher. Both atherosclerosis and reduced lung function develop slowly over the life-course and the cross-sectional design precludes any analysis of the temporal associations between them.

The participation rate was approximately 50% in this study. A recent study of non-participation in the SCAPIS study concluded that the selection bias appears to be small with respect to the risk factor distributions in the cohort [39]. However, generalizability to populations of other ages, ethnicities, and environments, is unknown.

## Conclusions

Reduced lung function is associated with subclinical atherosclerosis in the general population. This association is largely explained by higher prevalence of major cardiovascular risk factors in individuals with poor lung function. We found no evidence for a causal relationship between lung function and subclinical atherosclerosis. Assessing lung function in addition to conventional cardiovascular risk factors to gauge potential for detection of subclinical atherosclerosis is probably not meaningful, but a low lung function found by chance should alert for atherosclerosis.

### Supplementary Information

Below is the link to the electronic supplementary material.

Supplementary file1 (DOCX 89 kb)

Supplementary file2 (XLSX 158 kb)

Supplementary file2 (ppt 158 kb)

## Data Availability

The SCAPIS cohort is open for research applications, but restrictions could apply for legal reasons. More information can be found on the website (www.scapis.org).

## Abbreviations

CCTA: Coronary computed tomography angiography
D_LCO_: Diffusing capacity of lung for carbon monoxide
FEV_1_: Forced expiratory volume in 1 s
FVC: Forced vital capacity
MR: Mendelian randomization
IMT: Intima-media thickness
PAD: Peripheral artery disease
