# Supplementary material for: Pulmonary function and atherosclerosis in the general population: causal associations and clinical implications
Source: Eur J Epidemiol. 2024 Jan 2;39(1):35–49. doi: 10.1007/s10654-023-01088-z (PMC10811042; doi:10.1007/s10654-023-01088-z)
Supplement: Supplementary file 1 — Supplementary file1 (DOCX 89 kb) [file 10654_2023_1088_MOESM1_ESM.docx]

# Online resources:

Pulmonary function and atherosclerosis in the general population: Causal associations and clinical implications. European Journal of Epidemiology

Authors Gunnar Engström (MD, PhD)^a^, Erik Lampa (PhD)^b^, Koen Dekkers (MSc)^c^, Yi-Ting Lin (MD, PhD)^b, d, e^, Kristin Ahlm (PhD)^f^, Håkan Ahlström (MD, PhD)^g, h, i^, Joakim Alfredsson (MD, PhD)^j^, Göran Bergström (MD, PhD)^k, l^, Anders Blomberg (MD, PhD)^f^, John Brandberg (MD, PhD)^m, n^, Kenneth Caidahl (MD, PhD)^o, p^, Kerstin Cederlund (MD, PhD)^q^, Olov Duvernoy (MD, PhD)^g^, Jan E. Engvall (MD, PhD)^r, s^, Maria J Eriksson (MD, PhD)^o, t^, Tove Fall (PhD)^c^, Bruna Gigante (MD, PhD)^u, v^, Anders Gummesson (MD, PhD)^k, w^, Emil Hagström (MD, PhD)^x, y^, Viktor Hamrefors (MD, PhD)^a, z^, Jan Hedner (MD, PhD)^aa, ab^, Magnus Janzon (MD, PhD)^ac^, Tomas Jernberg (MD, PhD)^ad^, Linda Johnson (PhD)^a^, Lars Lind (MD, PhD)^ae^, Eva Lindberg (MD, PhD)^af^, Maria Mannila (MD, PhD)^ag^, Ulf Nilsson (MD, PhD)^f^, Anders Persson (MD, PhD)^r, ah, ai^, Hans Lennart Persson (MD, PhD)^aj^, Margaretha Persson (MPH, PhD)^a, ak^, Anna Ramnemark (MD, PhD)^al^, Annika Rosengren (MD, PhD)^k, am^, Caroline Schmidt (MD, PhD)^k^, Linn Skoglund Larsson (MD)^an^, C. Magnus Sköld (MD, PhD)^ao, ap^, Eva Swahn (MD, PhD)^ac^, Stefan Söderberg (MD, PhD)^f^, Kjell Torén (MD, PhD)^aq, ar^, Anders Waldenström (MD, PhD)^f^, Per Wollmer (MD, PhD)^as^, Suneela Zaigham (MBSS, PhD)^a, b^, Carl Johan Östgren (MD, PhD)^r, at^, Johan Sundström (MD, PhD)^b, au^

^a^Department of Clinical Sciences in Malmö, Lund University, Lund, Sweden; ^b^Department of Medical Sciences, Uppsala University, Uppsala, Sweden; ^c^Department of Medical Sciences, Molecular Epidemiology and Science for Life Laboratory, Uppsala University, Uppsala, Sweden; ^d^Department of Neurobiology, Care Sciences and Society, Karolinska Institute, Huddinge, Sweden; ^e^Department of Family Medicine, Kaohsiung Medical University, Kaohsiung City, Taiwan; ^f^Department of Public Health and Clinical Medicine, Section of Medicine, Umeå University, Umeå, Sweden; ^g^Department of Surgical Sciences, Section of Radiology, Uppsala University, Uppsala, Sweden; ^h^BFC, Uppsala University Hospital, Uppsala, Sweden; ^i^Antaros Medical AB, Mölndal, Sweden; ^j^Department of Cardiology, and Department of Health, Medicine and Caring Sciences, Unit of Cardiovascular Sciences, Linköping University Linköping, Sweden; ^k^Department of Molecular and Clinical Medicine, Institute of Medicine, Sahlgrenska Academy, University of Gothenburg, Gothenburg, Sweden; ^l^Clinical Physiology, Sahlgrenska University Hospital, Gothenburg, Sweden; ^m^Region Västra Götaland, Sahlgrenska University Hospital, Department of Radiology, Gothenburg, Sweden; ^n^Department of Radiology, Institute of Clinical Sciences, Sahlgrenska Academy, University of Gothenburg, Gothenburg, Sweden; ^o^Department of Clinical Physiology, Karolinska University Hospital, Stockholm, Sweden; ^p^Department of Clinical Physiology, Sahlgrenska University Hospital, and Sahlgrenska Academy, Gothenburg, Sweden; ^q^Department of Clinical Science, Intervention and Technology, Karolinska Institutet, Stockholm, Sweden; ^r^CMIV, Centre of Medical Image Science and Visualization, Linköping University, Linköping, Sweden; ^s^Department of Clinical Physiology, and Department of Health, Medicine and Caring Sciences, Linköping University, Linköping, Sweden; ^t^Department of Molecular Medicine and Surgery, Karolinska Institutet, Stockholm; ^u^Division of Cardiovascular Medicine Unit, Department of Medicine Solna, Karolinska Institute, Stockholm, Sweden; ^v^Department of Clinical Science, Danderyd University Hospital, Stockholm, Sweden; ^w^Department of Clinical Genetics and Genomics, Region Västra Götaland, Sahlgrenska University Hospital, Gothenburg, Sweden; ^x^Department of Medical Sciences, Cardiology, Uppsala University, Uppsala, Sweden; ^y^Uppsala Clinical Research Center, Uppsala University, Uppsala, Sweden; ^z^Department of Cardiology, Skåne University Hospital, Malmö, Sweden; ^aa^Pulmonary Department, Sleep Disorders Center, Sahlgrenska University Hospital, Gothenburg, Sweden; ^ab^Center of Sleep and Wake Disorders, Sahlgrenska Academy, Gothenburg University, Göteborg, Sweden; ^ac^Department of Cardiology, and Department of Health, Medicine and Caring Sciences, Unit of Cardiovascular Sciences, Linköping University, Linköping, Sweden; ^ad^Department of Clinical Sciences, Danderyd University Hospital, Karolinska Institutet, Stockholm, Sweden; ^ae^Department of Medical Sciences, Clinical Epidemiology, Uppsala University, Uppsala, Sweden; ^af^Department of Medical Sciences, Respiratory, Allergy and Sleep Research, Uppsala University, Sweden; ^ag^Heart and Vascular Theme, Department of Cardiology, and Clinical Genetics, Karolinska University Hospital, Stockholm, Sweden; ^ah^Department of Radiology, and Department of Health, Medicine and Caring Sciences, Linköping University, Linköping, Sweden; ^ai^Department of Clinical Sciences, Huddinge University Hospital, Karolinska Institute, Stockholm, Sweden
^aj^Respiratory Medicine, Department of Medical and Health Sciences (IMH), Linköping University, Linköping, Sweden; ^ak^Department of Internal Medicine, Skåne University Hospital, Malmö, Sweden; ^al^Department of Community Medicine and Rehabilitation, Geriatric Medicine, Umeå university, Umeå, Sweden; ^am^Department of Medicine Geriatrics and Emergency Medicine, Sahlgrenska University Hospital Östra Hospital, Gothenburg, Sweden; ^an^Department of Public Health and Clinical Medicine, Umeå University, Umeå, Sweden; ^ao^Department of Respiratory Medicine and Allergy, Karolinska University Hospital Solna, Stockholm, Sweden; ^ap^Respiratory Medicine Unit, Department of Medicine Solna and Center for Molecular Medicine, Karolinska Institutet, Stockholm, Sweden; ^aq^Section of Occupational and Environmental Medicine, School of Public Health and Community Medicine, Institute of Medicine, Sahlgrenska Academy, University of Gothenburg, Gothenburg, Sweden; ^ar^Department of Occupational and Environmental Medicine, Sahlgrenska University Hospital, Gothenburg, Sweden; ^as^Department of Translational Medicine, Lund University, Malmö; ^at^Department of Health, Medicine and Caring Sciences, Linköping University, Linköping, Sweden
^au^The George Institute for Global Health, University of New South Wales, Sydney, Australia

Corresponding author: Gunnar Engström, Department of Clinical science in Malmö, Lund university, Sweden. Email: [Gunnar.Engstrom@med.lu.se](mailto:Gunnar.Engstrom@med.lu.se).

**TABLE OF CONTENTS**

Legends to figures, online resources p 4

Table 1. Distribution of risk factors in relation to quintiles of FEV_1_ in men. P 5

Table 2. Distribution of risk factors in relation to quintiles of FEV_1_ in women p 7

Table 3. Distribution of risk factors in relation to quintiles of D_LCO_ in men. P 9

Table 4. Distribution of risk factors in relation to quintiles of D_LCO_ in women. P 11

Online Methods, Mendelian Randomization p 13

SCAPIS Study Organization p 16

# Legends to Online resource figures

## Online resource Figure 1. Directed acyclic graph of assumed causal relationships between lung function and atherosclerosis. Age, sex, height, site, smoking, obesity (waist circumference), diabetes, physical activity, inflammation (CRP), and socioeconomic status (education) were considered potential confounders and therefore included in the multivariate analysis. Cholesterol and blood pressure are known risk factors for atherosclerosis but are unlikely to have any effect on lung function (independently of atherosclerosis) and were therefore not included in the multivariate model.

Online resource Figure 2. Distribution of FEV_1_/FVC <0.7, any CT-assessed emphysema, and atherosclerosis in three vascular beds (CACS>0, carotid plaque and ABI<0.9) in men (left figure) and women (right figure)

Online resource Figure 3a and 3b. Forest plots of men (3a) and women (3b) adjusted for height, age and site. Adjusted OR (95%Cis) for a sex-specific interquartile range increase in the exposures except for the Emphysema score where the OR corresponds to an increase between zero and one. "Low" and "high" indicate the interquartile range for the exposure variables.

Online resource Figure 4. Associations of lung function and emphysema score with atherosclerosis in three arterial beds **in never smokers**. Associations between exposures (columns) and outcomes (rows) in men (red) and women (blue). Depicted are the probability of SIS >=2 (top row, A), probability of CACS > 0 (B), probability of ABI < 0.9 (C) and the probability of plaque in at least one coronary artery (D). Curves are truncated at the sex-specific 10^th and 90^th percentiles of the exposure variables and vertical broken lines indicate the sex specific interquartile range (IQR) except for the Emphysema score where the IQR equals zero and lines are drawn at zero and one.

| Online resource Table 1. Distribution of risk factors in relation to quintiles of FEV_1_ in men. | | | | |  |  |
| --- | --- | --- | --- | --- | --- | --- |
| **Quintiles of FEV_1_, Men** | **1** | | **2** | **3** | **4** | **5** |
| FEV_1_ (L) | | <3.30 | 3.30-3.67 | 3.67-3.98 | 3.98-4.34 | >4.34 |
| N | 2859 | | 2859 | 2859 | 2859 | 2858 |
| Age (years) | 60.3 [56.4, 63.0] | | 58.6 [54.6, 61.8] | 57.7 [53.8, 61.3] | 56.4 [53.1, 60.2] | 54.8 [52.2, 58.4] |
| Height (cm) | 174 [170, 179] | | 177 [173, 181] | 179 [175, 183] | 181 [178, 185] | 184 [181, 188] |
| Weight (kg) | 84.5 [76.0, 94.8] | | 85.0 [77.8, 94.7] | 86.5 [79.0, 95.4] | 87.0 [80.0, 96.2] | 89.6 [82.4, 98.3] |
| BMI (kg/m^2^) | 27.7 [25.3, 30.8] | | 27.2 [25.0, 30.1] | 26.9 [24.8, 29.4] | 26.6 [24.5, 29.0] | 26.3 [24.4, 28.6] |
| Smoking status (%) |  | |  |  |  |  |
| Current smoker | 582 (21.2) | | 374 (13.6) | 313 (11.4) | 249 (9.0) | 238 (8.6) |
| Former smoker | 1043 (38.0) | | 1018 (36.9) | 908 (33.0) | 910 (32.9) | 773 (28.0) |
| Never smoker | 1120 (40.8) | | 1367 (49.5) | 1528 (55.6) | 1603 (58.0) | 1752 (63.4) |
| College or University degree (%) | 954 (34.7) | | 1085 (39.2) | 1078 (38.9) | 1195 (42.9) | 1351 (48.2) |
| Systolic BP (mmHg) | 131 [120, 142] | | 128 [118, 139] | 127 [118, 138] | 126 [117, 136] | 124 [116, 134] |
| Diastolic BP (mmHg) | 79 [73, 86] | | 78 [72, 85] | 78 [72, 85] | 78 [71, 84] | 76 [70, 83] |
| Anti-hypertensive medication (%) | 855 (31.5) | | 661 (24.1) | 557 (20.3) | 496 (17.9) | 391 (14.1) |
| LDL cholesterol (mmol/L) | 3.3 [2.7, 4.0] | | 3.4 [2.8, 4.0] | 3.4 [2.8, 4.1] | 3.4 [2.8, 4.1] | 3.4 [2.8, 4.0] |
| HDL cholesterol (mmol/L) | 1.3 [1.1, 1.5] | | 1.3 [1.1, 1.6] | 1.4 [1.2, 1.6] | 1.4 [1.2, 1.7] | 1.4 [1.2, 1.7] |
| Lipid-lowering medication (%) | 406 (15.0) | | 324 (11.8) | 254 (9.2) | 218 (7.9) | 161 (5.8) |
| Diabetes (%) | 500 (17.5) | | 322 (11.3) | 243 (8.5) | 186 (6.5) | 115 (4.0) |
| CRP (mg/L) | 1.5 [0.80, 3.1] | | 1.1 [0.60, 2.2] | 1.0 [0.60, 1.9] | 0.90 [0.60, 1.7] | 0.80 [0.60, 1.4] |
| Sedentary time (% of day) | 57 [49, 64] | | 57 [49, 63] | 56 [49, 63] | 57 [49, 63] | 57 [49, 63] |
| FVC (liter) | 3.97 [3.66, 4.30] | | 4.49 [4.26, 4.74] | 4.87 [4.65, 5.13] | 5.24 [5.01, 5.50] | 5.83 [5.54, 6.20] |
| FEV_1_/FVC | 0.75 [0.69, 0.80] | | 0.78 [0.74, 0.82] | 0.78 [0.75, 0.82] | 0.79 [0.76, 0.82] | 0.80 [0.76, 0.83] |
| D_LCO_ (mmol/(min kPa)) | 8.59 [7.61, 9.54] | | 9.38 [8.54, 10.3] | 9.88 [9.00, 10.8] | 10.3 [9.45, 11.2] | 11.0 [10.1, 12.0] |
| ABI | 1.21 [1.15, 1.27] | | 1.23 [1.17, 1.29] | 1.23 [1.17, 1.29] | 1.24 [1.18, 1.30] | 1.24 [1.18, 1.30] |
| ABI < 0.9 (%) | 29 (1.0) | | 15 (0.5) | 1 (0.0) | 2 (0.1) | 0 (0.0) |
| Segment Involvement Score >= 2 (%) | 592 (32.1) | | 533 (26.4) | 458 (21.8) | 421 (19.4) | 361 (16.4) |
| CACS category n (%) |  | |  |  |  |  |
| 0 | 973 (36.0) | | 1108 (40.5) | 1306 (47.1) | 1405 (50.3) | 1458 (52.0) |
| 1-99 | 1002 (37.1) | | 1041 (38.0) | 966 (34.9) | 961 (34.4) | 966 (34.4) |
| >=100 | 728 (26.9) | | 590 (21.5) | 498 (18.0) | 429 (15.3) | 381 (13.6) |

Values are medians [Interquartile range] or n (%)

| Online resource Table 2. Distribution of risk factors in relation to quintiles of FEV_1_ in women. | | | | |  |  |
| --- | --- | --- | --- | --- | --- | --- |
| **Quintiles of FEV_1_, Women** | **1** | | **2** | **3** | **4** | **5** |
| FEV_1_ (L) | | <2.39 | 2.39-2.64 | 2.64-2.86 | 2.86-3.13 | >3.13 |
| N | 3007 | | 3007 | 3007 | 3007 | 3006 |
| Age (years) | 60.3 [56.6, 62.9] | | 58.8 [55.0, 62.0] | 57.6 [54.2, 61.1] | 56.2 [53.0, 59.9] | 54.5 [52.1, 58.0] |
| Height (cm) | 161 [157, 165] | | 164 [160, 167] | 165 [162, 169] | 167 [164, 171] | 171 [167, 174] |
| Weight (kg) | 69.9 [61.6, 80.3] | | 70.0 [62.0, 79.8] | 69.8 [62.8, 79.4] | 70.5 [63.9, 79.1] | 72.0 [65.0, 81.0] |
| BMI (kg/m^2^) | 26.9 [23.9, 31.0] | | 26.1 [23.3, 29.6] | 25.5 [22.9, 29.0] | 25.2 [22.9, 28.3] | 24.8 [22.5, 27.7] |
| Smoking status (%) |  | |  |  |  |  |
| Current smoker | 556 (19.1) | | 418 (14.4) | 306 (10.4) | 304 (10.4) | 282 (9.7) |
| Former smoker | 1181 (40.6) | | 1125 (38.6) | 1192 (40.7) | 1127 (38.6) | 1088 (37.3) |
| Never smoker | 1175 (40.4) | | 1369 (47.0) | 1431 (48.9) | 1490 (51.0) | 1549 (53.1) |
| College or University degree (%) | 1121 (38.5) | | 1341 (45.5) | 1533 (51.8) | 1574 (53.2) | 1686 (57.1) |
| Systolic BP (mmHg) | 126 [114, 138] | | 122 [112, 135] | 121 [110, 134] | 119 [109, 132] | 118 [108, 128] |
| Diastolic BP (mmHg) | 78 [70, 85] | | 76 [70, 84] | 76 [69, 84] | 76 [68, 83] | 74 [68, 82] |
| Anti-hypertensive medication (%) | 748 (25.9) | | 622 (21.2) | 491 (16.8) | 449 (15.3) | 353 (12.0) |
| LDL cholesterol (mmol/L) | 3.5 [2.8, 4.1] | | 3.5 [2.8, 4.1] | 3.4 [2.8, 4.1] | 3.4 [2.8, 4.0] | 3.2 [2.7, 3.8] |
| HDL cholesterol (mmol/L) | 1.7 [1.4, 2.0] | | 1.8 [1.4, 2.1] | 1.8 [1.5, 2.2] | 1.8 [1.5, 2.2] | 1.8 [1.6, 2.2] |
| Lipid-lowering medication (%) | 295 (10.2) | | 213 (7.3) | 150 (5.1) | 131 (4.5) | 97 (3.3) |
| Diabetes (%) | 294 (9.8) | | 185 (6.2) | 146 (4.9) | 123 (4.1) | 73 (2.4) |
| CRP (mg/L) | 1.6 [0.80, 3.4] | | 1.02 [0.60, 2.6] | 1.0 [0.60, 2.2] | 0.80 [0.60, 1.9] | 0.70 [0.60, 1.5] |
| Sedentary time (% of day) | 53 [45, 60] | | 52 [45, 59] | 53 [45, 59] | 53 [46, 59] | 53 [46, 59] |
| FVC (liter) | 2.82 [2.61, 3.04] | | 3.20 [3.05, 3.38] | 3.45 [3.32, 3.64] | 3.74 [3.59, 3.92] | 4.20 [3.99, 4.45] |
| FEV_1_/FVC | 0.77 [0.71, 0.81] | | 0.79 [0.75, 0.82] | 0.79 [0.76, 0.82] | 0.80 [0.76, 0.83] | 0.80 [0.77, 0.83] |
| D_LCO_ (mmol/(min kPa)) | 6.40 [5.70, 7.10] | | 6.89 [6.27, 7.54] | 7.21 [6.60, 7.90] | 7.54 [6.89, 8.23] | 8.02 [7.34, 8.79] |
| ABI | 1.16 [1.10, 1.23] | | 1.18 [1.12, 1.25] | 1.20 [1.13, 1.26] | 1.20 [1.14, 1.26] | 1.21 [1.15, 1.27] |
| ABI < 0.9 (%) | 14 (0.5) | | 4 (0.1) | 4 (0.1) | 3 (0.1) | 2 (0.1) |
| Segment Involvement Score >= 2 (%) | 225 (11.3) | | 170 (7.9) | 160 (7.1) | 122 (5.3) | 107 (4.6) |
| CACS category n (%) |  | |  |  |  |  |
| 0 | 1849 (62.9) | | 2047 (68.9) | 2212 (74.2) | 2230 (74.9) | 2352 (78.9) |
| 1-99 | 786 (26.7) | | 716 (24.1) | 611 (20.5) | 625 (21.0) | 526 (17.6) |
| >=100 | 305 (10.4) | | 210 (7.1) | 159 (5.3) | 122 (4.1) | 104 (3.5) |

| Online resource Table 3. Distribution of risk factors in relation to quintiles of D_LCO_ in men. | | | | |  |  |
| --- | --- | --- | --- | --- | --- | --- |
| **Quintiles of D_LCO_, Men** | **1** | | **2** | **3** | **4** | **5** |
| D_LCO_ (mmol/(min kPa)) | | <8.56 | 8.56-9.47 | 9.47-10.3 | 10.3-11.2 | >11.2 |
| N | 2781 | | 2781 | 2781 | 2780 | 2780 |
| Age (years) | 60.3 [56.3, 63.0] | | 58.6 [54.6, 61.9] | 57.2 [53.6, 60.9] | 56.3 [52.9, 60.2] | 55.2 [52.4, 58.8] |
| Height (cm) | 175 [171, 180] | | 178 [174, 182] | 179 [176, 184] | 181 [177, 185] | 184 [179, 188] |
| Weight (kg) | 83.0 [75.0, 92.0] | | 85.1 [78.0, 94.0] | 87.0 [79.2, 96.6] | 88.0 [80.5, 97.5] | 90.0 [82.7, 99.4] |
| BMI (kg/m^2^) | 27.0 [24.7, 29.7] | | 27.1 [24.9, 29.6] | 26.94 [24.79, 29.56] | 26.9 [24.8, 29.5] | 26.7 [24.6, 29.4] |
| Smoking status (%) |  | |  |  |  |  |
| Current smoker | 687 (25.7) | | 347 (13.0) | 255 (9.5) | 235 (8.7) | 161 (6.0) |
| Former smoker | 987 (36.9) | | 972 (36.4) | 916 (34.3) | 871 (32.3) | 776 (28.8) |
| Never smoker | 998 (37.4) | | 1348 (50.5) | 1501 (56.2) | 1591 (59.0) | 1760 (65.3) |
| College or University degree (%) | 941 (35.2) | | 1043 (38.9) | 1120 (41.6) | 1148 (42.2) | 1278 (46.7) |
| Systolic BP (mmHg) | 128 [118, 138] | | 126 [117, 138] | 126 [118, 138] | 126 [118, 138] | 127 [118, 138] |
| Diastolic BP (mmHg) | 78 [72, 84] | | 78 [71, 84] | 78 [71, 85] | 78 [72, 85] | 78 [72, 85] |
| Anti-hypertensive medication (%) | 763 (28.8) | | 650 (24.6) | 549 (20.5) | 502 (18.7) | 414 (15.3) |
| LDL cholesterol (mmol/L) | 3.30 [2.60, 4.00] | | 3.50 [2.80, 4.10] | 3.50 [2.80, 4.10] | 3.40 [2.80, 4.10] | 3.40 [2.80, 4.00] |
| HDL cholesterol (mmol/L) | 1.30 [1.10, 1.60] | | 1.30 [1.10, 1.60] | 1.40 [1.10, 1.60] | 1.40 [1.20, 1.60] | 1.40 [1.20, 1.70] |
| Lipid-lowering medication (%) | 409 (15.5) | | 300 (11.3) | 235 (8.8) | 219 (8.2) | 156 (5.8) |
| Diabetes (%) | 450 (16.2) | | 295 (10.6) | 210 (7.6) | 196 (7.1) | 170 (6.1) |
| CRP (mg/L) | 1.4 [0.70, 1.9] | | 1.1 [0.60, 2.1] | 1.0 [0.60, 1.9] | 0.90 [0.60, 1.8] | 0.80 [0.60, 1.5] |
| Sedentary time (% of day) | 57 [49, 64] | | 56 [49, 63] | 56 [49, 63] | 56 [49, 63] | 56 [49, 63] |
| FEV_1_ (liter) | 3.32 [2.96, 3.68] | | 3.65 [3.31, 3.98] | 3.86 [3.52, 4.21] | 3.99 [3.66, 4.34] | 4.25 [3.88, 4.63] |
| FVC (liter) | 4.30 [3.85, 4.76] | | 4.68 [4.26, 5.11] | 4.94 [4.51, 5.37] | 5.12 [4.71, 5.59] | 5.49 [5.06, 6.00] |
| FEV_1_/FVC | 0.78 [0.72, 0.82] | | 0.79 [0.75, 0.82] | 0.79 [0.75, 0.82] | 0.78 [0.74, 0.82] | 0.78 [0.74, 0.81] |
| ABI | 1.21 [1.15, 1.28] | | 1.23 [1.17, 1.29] | 1.23 [1.18, 1.29] | 1.23 [1.17, 1.30] | 1.23 [1.17, 1.29] |
| ABI < 0.9 (%) | 28 (1.0) | | 6 (0.2) | 6 (0.2) | 1 (0.0) | 1 (0.0) |
| Segment Involvement Score >= 2 (%) | 566 (31.3) | | 487 (24.9) | 456 (22.3) | 416 (19.9) | 376 (17.3) |
| CACS category n (%) |  | |  |  |  |  |
| 0 | 948 (36.0) | | 1160 (43.6) | 1237 (45.7) | 1333 (49.2) | 1420 (51.8) |
| 1-99 | 942 (35.8) | | 967 (36.4) | 995 (36.7) | 950 (35.1) | 942 (34.3) |
| >=100 | 740 (28.1) | | 533 (20.0) | 476 (17.6) | 426 (15.7) | 381 (13.9) |

| Online resource Table 4. Distribution of risk factors in relation to quintiles of D_LCO_ in women. | | | | |  |  |
| --- | --- | --- | --- | --- | --- | --- |
| **Quintiles of D_LCO_, Women** | **1** | | **2** | **3** | **4** | **5** |
| D_LCO_ (mmol/(min kPa)) | | <6.31 | 6.31-6.94 | 6.94-7.52 | 7.52-8.22 | >8.22 |
| N | 2926 | | 2926 | 2925 | 2925 | 2925 |
| Age (years) | 59.8 [55.9, 62.7] | | 58.4 [54.7, 61.7] | 57.4 [53.9, 61.1] | 56.6 [53.1, 60.4] | 55.1 [52.3, 58.7] |
| Height (cm) | 162 [158, 166] | | 164 [160, 168] | 165 [162, 169] | 167 [163, 171] | 169 [165, 174] |
| Weight (kg) | 69.0 [61.0, 78.0] | | 69.9 [62.7, 79.0] | 70.2 [63.0, 79.8] | 70.7 [64.0, 80.6] | 72.4 [65.0, 81.3] |
| BMI (kg/m^2^) | 26.2 [23.3, 29.8] | | 25.8 [23.3, 29.3] | 25.6 [23.0, 29.1] | 25.4 [22.9, 28.9] | 25.1 [22.7, 28.4] |
| Smoking status (%) |  | |  |  |  |  |
| Current smoker | 723 (25.6) | | 371 (13.0) | 293 (10.3) | 236 (8.3) | 179 (6.3) |
| Former smoker | 1101 (39.0) | | 1197 (42.1) | 1152 (40.4) | 1112 (39.0) | 1032 (36.4) |
| Never smoker | 1000 (35.4) | | 1275 (44.8) | 1409 (49.4) | 1500 (52.7) | 1622 (57.3) |
| College or University degree (%) | 1127 (39.7) | | 1335 (46.6) | 1449 (50.4) | 1499 (52.1) | 1663 (57.7) |
| Systolic BP (mmHg) | 122 [111, 134] | | 121 [110, 134] | 120 [110, 134] | 121 [110, 134] | 120 [111, 133] |
| Diastolic BP (mmHg) | 76 [69, 83] | | 76 [69, 84] | 76 [69, 84] | 76 [69, 84] | 76 [69, 84] |
| Anti-hypertensive medication (%) | 704 (25.1) | | 544 (19.2) | 492 (17.2) | 447 (15.6) | 391 (13.6) |
| LDL cholesterol (mmol/L) | 3.5 [2.8, 4.2] | | 3.5 [2.8, 4.1] | 3.4 [2.8, 4.1] | 3.4 [2.8, 4.0] | 3.2 [2.7, 3.8] |
| HDL cholesterol (mmol/L) | 1.7 [1.4, 2.0] | | 1.8 [1.5, 2.1] | 1.8 [1.5, 2.1] | 1.8 [1.5, 2.2] | 1.9 [1.6, 2.2] |
| Lipid-lowering medication (%) | 310 (11.0) | | 182 (6.4) | 143 (5.0) | 126 (4.4) | 99 (3.4) |
| Diabetes (%) | 239 (8.2) | | 175 (6.0) | 151 (5.2) | 127 (4.3) | 102 (3.5) |
| CRP (mg/L) | 1.4 [0.70, 3.1] | | 1.2 [0.60, 2.5] | 1.1 [0.60, 2.4] | 0.90 [0.60, 1.9] | 0.80 [0.60, 1.6] |
| Sedentary time (% of day) | 54 [46, 60] | | 52 [45, 59] | 53 [46, 59] | 52 [45, 59] | 53 [45, 59] |
| FEV_1_ (liter) | 2.41 [2.14, 2.67] | | 2.64 [2.40, 2.90] | 2.75 [2.51, 3.01] | 2.87 [2.61, 3.14] | 3.06 [2.79, 3.36] |
| FVC (liter) | 3.10 [2.78, 3.43] | | 3.36 [3.04, 3.68] | 3.49 [3.19, 3.80] | 3.65 [3.33, 3.97] | 3.90 [3.56, 4.27] |
| FEV_1_/FVC | 0.78 [0.74, 0.82] | | 0.79 [0.75, 0.83] | 0.79 [0.76, 0.83] | 0.79 [0.76, 0.82] | 0.79 [0.75, 0.82] |
| ABI | 1.17 [1.11, 1.24] | | 1.19 [1.12, 1.25] | 1.19 [1.14, 1.26] | 1.20 [1.14, 1.26] | 1.20 [1.14, 1.26] |
| ABI < 0.9 (%) | 17 (0.6) | | 6 (0.2) | 2 (0.1) | 1 (0.0) | 1 (0.0) |
| Segment Involvement Score >= 2 (%) | 227 (11.4) | | 171 (8.0) | 139 (6.5) | 118 (5.4) | 106 (4.7) |
| CACS category n (%) |  | |  |  |  |  |
| 0 | 1807 (63.1) | | 2064 (71.2) | 2136 (73.8) | 2170 (74.8) | 2229 (77.1) |
| 1-99 | 754 (26.3) | | 658 (22.7) | 633 (21.9) | 583 (20.1) | 553 (19.1) |
| >=100 | 304 (10.6) | | 175 (6.0) | 126 (4.4) | 149 (5.1) | 110 (3.8) |

## Online methods, Mendelian randomization

Mendelian randomization (MR) is a method that uses genetic variants as causal anchors to obtain causal estimates for observational associations in the presence of potential confounding. It has been compared to randomized control trials, where instead of the treatment being randomized, the genetic variants have been randomized according to Mendel’s law^1–3^. One of the potential pitfalls of MR is horizontal pleiotropy, however many methods have been developed to counteract this^4–7^.

All MR analyses were performed using R (version 4.1.1). In the primary MR analysis, genetic variants associated with FEV_1_, FVC and FEV_1_/FVC were used as genetic instruments to assess the causal effect of lung function on atherosclerosis (carotid IMT, presence of carotid plaques, PAD). Genetic variants associated with carotid IMT and PAD were used as genetic instruments to assess the causal effect of atherosclerosis on lung function (FEV_1_, FVC, FEV_1_/FVC). Since only 5 genetic variants were independently associated with presence of carotid plaques^9^, we decided to only assess the causal effect of lung function on presence of carotid plaques and not vice versa.

The selected genetic instruments for lung function included 37, 43 and 55 variants independently associated (p-value < 5 x 10^-8^, not in LD) with FEV_1_, FVC and FEV_1_/FVC identified in UK Biobank (N = 321,047)^8^. The selected genetic instruments for atherosclerosis included 11 genetic variants independently associated with carotid IMT (N = 71,128), identified in the CHARGE consortium^9^, and 18 genetic variants independently associated with PAD (Cases = 31,307, Controls = 211,753), identified in the MVP consortium^10^.

In the secondary analysis, we used random effects MR-Egger, weighted-median MR and CAUSE MR to assess pleiotropic effects, and multivariable MR to specifically assess the potential effect of smoking and height.

In the tertiary analysis, we repeated the previous analyses in never-smokers where possible.

To obtain beta coefficients and standard errors for genetic variants associated with FVC, FEV_1_ and FEV_1_/FVC we performed GWAS in the UK Biobank (all participants, N = 255,647; never-smokers, N = 138,122; UK Biobank Application 52678). The GWAS were performed using plink v2.00-alpha-2-20190429 with the following thresholds: minor allele count = 30, variance inflation factor = 50, MACH-R2 imputation quality = 0.8 - 2, missing call rate (both variants and samples) = 0.1, Hardy-Weinberg equilibrium p-value = 10^-6^. The models were adjusted for age, sex, genotyping array and the first 20 genetic principal components. Beta coefficients and standard errors for carotid IMT and presence of carotid plaques were obtained from the CHARGE consortium (dbGaP phs000930) and for PAD from the MVP consortium (dbGaP phs001672). Beta coefficients and standard errors for smoking (N = 74,035) and height (N = 253,288) were obtained from the Tobacco and Genetics Consortium^11^ and the GIANT consortium^12^, respectively. Specifically for never-smokers, beta coefficients for carotid IMT were obtained using a GWAS in UK Biobank using the same settings as above, with carotid IMT defined as the mean of the four available measurements (N = 10,951). For each GWAS, the beta coefficients were scaled by their standard deviation and the standard errors were recalculated from the scaled beta coefficients and p-values.

For the random effects inverse-variance weighted, random effects MR-Egger, and weighted median MR analyses, genetic proxies were obtained using the get_ld_proxies function of the genetics.binaRies v0.0.0.9000 package based on correlation data of European genomes of the 1000 Genomes project^13^, genetic variants were harmonized using the harmonize_data function of the TwoSampleMR^14^ v0.5.4 package, and MR was performed using the mr_input and mr_allmethods functions of the MendelianRandomization^15^ v0.5.1 package. For the multivariable MR analysis, the same procedure was followed, but the mr_mvinput and mr_mvivw functions of the MendelianRandomization package were used. For the CAUSE MR analysis, the cause v1.2.0 package^5^ was used: genetic variants were harmonized using the gwas_merge function, parameters were estimated using the est_cause_params function on a random subset of 1000000 genetic variants, genetic variants were clumped using the ld_clump function using correlation data of the European genomes of the 1000 Genomes project with a p-value cutoff of 0.001, and MR was performed using the cause function.

The number of genetic variants available for analysis, i.e. available in exposure and outcome GWAS or with a genetic proxy, was at least 34 out of 37 for FEV_1_, 41 out of 43 for FVC, 51 out of 55 for FEV_1_/FVC, 7 out of 11 for carotid IMT and 16 out of 18 for PAD. All genetic instruments were valid for use in MR (median F-statistics of genetic variants > 10; Online resources Table 5).

## Online resource references

1. Burgess, S. *et al.* Guidelines for performing Mendelian randomization investigations. *Wellcome Open Res* **4**, 186 (2020).

2. Davey Smith, G. & Hemani, G. Mendelian randomization: genetic anchors for causal inference in epidemiological studies. *Hum Mol Genet* **23**, R89–R98 (2014).

3. Davies, N. M., Holmes, M. V. & Smith, G. D. Reading Mendelian randomisation studies: a guide, glossary, and checklist for clinicians. *BMJ* **362**, k601 (2018).

4. Bowden, J., Davey Smith, G. & Burgess, S. Mendelian randomization with invalid instruments: effect estimation and bias detection through Egger regression. *Int J Epidemiol* **44**, 512–525 (2015).

5. Morrison, J., Knoblauch, N., Marcus, J. H., Stephens, M. & He, X. Mendelian randomization accounting for correlated and uncorrelated pleiotropic effects using genome-wide summary statistics. *Nat Genet* **52**, 740–747 (2020).

6. Burgess, S. & Thompson, S. G. Multivariable Mendelian Randomization: The Use of Pleiotropic Genetic Variants to Estimate Causal Effects. *Am J Epidemiol* **181**, 251–260 (2015).

7. Bowden, J., Smith, G. D., Haycock, P. C. & Burgess, S. Consistent Estimation in Mendelian Randomization with Some Invalid Instruments Using a Weighted Median Estimator. *Genetic Epidemiology* **40**, 304–314 (2016).

8. Shrine, N. *et al.* New genetic signals for lung function highlight pathways and chronic obstructive pulmonary disease associations across multiple ancestries. *Nat Genet* **51**, 481–493 (2019).

9. Franceschini, N. *et al.* GWAS and colocalization analyses implicate carotid intima-media thickness and carotid plaque loci in cardiovascular outcomes. *Nat Commun* **9**, 5141 (2018).

10. Klarin, D. *et al.* Genome-wide association study of peripheral artery disease in the Million Veteran Program. *Nat Med* **25**, 1274–1279 (2019).

11. Furberg, H. *et al.* Genome-wide meta-analyses identify multiple loci associated with smoking behavior. *Nat Genet* **42**, 441–447 (2010).

12. Wood, A. R. *et al.* Defining the role of common variation in the genomic and biological architecture of adult human height. *Nat Genet* **46**, 1173–1186 (2014).

13. Auton, A. *et al.* A global reference for human genetic variation. *Nature* **526**, 68–74 (2015).

14. Hemani, G., Tilling, K. & Smith, G. D. Orienting the causal relationship between imprecisely measured traits using GWAS summary data. *PLOS Genetics* **13**, e1007081 (2017).

15. Yavorska, O. O. & Burgess, S. MendelianRandomization: an R package for performing Mendelian randomization analyses using summarized data. *International Journal of Epidemiology* **46**, 1734–1739 (2017).

# SCAPIS Study Organization

## Study governance (active and former)

### National Steering Committee

#### Directors:

Göran Bergström, University of Gothenburg, Director

Carl Johan Östgren, Linköping University, vice Director

#### Members (active and former):

Anders Blomberg, Umeå University, Umeå; John Brandberg, University of Gothenburg, Gothenburg; Kerstin Cederlund, Karolinska Institutet, Stockholm; Gunnar Engström, Lund University, Lund; Jan Engvall, Linköping University, Linköping; Mats G. Hansson, Uppsala University, Uppsala; Tomas Jernberg, Karolinska Institutet, Stockholm; Lars Lind, Uppsala University, Uppsala; Eva Lindberg,Uppsala University, Uppsala; Margaretha Persson, Lund University, Lund; Fredrik Nyström, Linköping University, Linköping; Annika Rosengren, University of Gothenburg, Gothenburg; Magnus Sköld,Karolinska Institutet, Stockholm; Johan Sundström, Uppsala University, Uppsala; Stefan Söderberg,Umeå University, Umeå; Kjell Torén, University of Gothenburg, Gothenburg

#### Coordinator:

Bim Boberg, the Swedish Heart and Lung Foundation, Stockholm

The Swedish CArdioPulmonary bioImage Study (SCAPIS) is a collaborative project between the following

Swedish Universities and Swedish University Hospitals:

University of Gothenburg and Sahlgrenska University Hospital; Karolinska Institutet and Karolinska University Hospital; Linköping University and Linköping University Hospital; Lund University and Skåne University Hospital; Umeå University and University Hospital of Umeå; Uppsala University and Uppsala University Hospital.

## Scientific Advisory Board

Marike Boezen, University of Groningen, Groningen, NL; Göran Berglund, Skåne University Hospital and Lund University, Malmö, SE; Robert Clarke, University of Oxford, UK; Marc Dewey, Radiology Institute at Charité University of Medicine, Berlin, GE; Ulf De Faire, Karolinska Institutet, Solna, SE; KayTee Khaw, University of Cambridge, UK; Juhani Knuuti, Turku PET Centre, Turku University Hospital, Turku, FI; Claes-Göran Löfdahl, Skåne University Hospital and Lund University, Lund, SE; Eva Prescott,University of Copenhagen and Bispebjerg University Hospital, Copenhagen, DK; Anders Waldenström,Umeå University, Umeå, SE

## Key persons involved in early discussions

Göran Berglund, Göran Bergström, Björn Fagerberg, Ulf de Faire, Jan Engvall, Bo Hedblad, Christer

Janson, Lars Johansson, Lars Lind, Claes-Göran Löfdahl, Fredrik Nyström, Anders Persson, Annika

Rosengren, Stefan Söderberg, Anders Waldenström, Hans Wedel

## Supportive Organization & Coordinating site

The Swedish Heart and Lung Foundation [2019-0012] is the main funder of SCAPIS. In addition to

providing financial support, the Swedish Heart and Lung Foundation has facilitated this national

collaborative project by assisting in arranging study meetings, providing travel support and meeting venues,

and sharing their expertise in communicating science.

### Swedish Heart and Lung Foundation

Kristina Sparreljung (secretary general), Staffan Josephsson (former secretary general), Mira Ernkvist, Bim

Boberg, Amanda Skog Andreasson, Ebba Bergman, Anna Fredholm, Louise Fornander, Susanne Klofsten,

Christa Larsvall, Anna Sjöström, Sofia Swedenborg, Joanna Tingström, Caroline Waldenström Sylvén,

Sofia Wirsén, Jan Nilsson, Anders Waldenström

### Study operations

#### Operational Management Team

Eva Karin Anderberg, Anna Andreasson, Göran Bergström, Anna Beskow, Bim Boberg, Charlotta Elfström, Mira Ernkvist, Peter Hedman, Carl Johan Östgren

#### SCAPIS Office

Eva Karin Anderberg (coordinator), Kristina Levan, Åsa Odhagen Rosvall, Charlotte Benninge

#### National Project Managers

National Project Coordinator: Charlotta Elfström

Project Managers: Sven Anders Benjegård, Ebba Bergman, Martin Brandhagen, Sven Burman, Olivia

Claesson, Louise Fornander, Anna Frick, Urban Gustafsson, Emma Larsson, Maria Matson Dzebo, Tina

Noord, Klara Thorsson

#### Data Management

IT Project Lead: Tina Noord (main coordinator), Anita Adolfsson, Anna Andreasson

Product Owner and Product Specialist: Anna Frick, Christian Johansen, Emma Larsson, Theodor Lewén

IT Development Team: Solution Architects: Christian Johansen, Tomas Snäckerström, Andreas Wallén; *System Developers*: Simon Dirnberger, Johan Fredin, Albin Willman, Max Jourdanis

Study Data Management: Niklas Svensson

Image Data Storage: Emanuel Hillberg, Nasser Hosseini, Åke Marjamäki, Tomas Moberg

Accelerometry Data Management: Örjan Ekblom, Elin Ekblom-Bak

Local IT Support: *Gothenburg*: Anders Broman, Johan Fors, Roger Lampa; *Linköping:* Tomas Annerholm;

*Malmö*: Pawel Gagol; *Umeå*: Gunnar Jonsson, Fredrik Lejon, Mattias Wennberg; *Uppsala*: Jörgen Anell,

Martin Karlsson, José Conde Herrera

#### Head Project Statistician

Erik Lampa

#### Publication Support

Ebba Bergman, Louise Fornander, Sofia Swedenborg, Ruby Rahman, Rebecca Josefson

#### Senior Scientific Editor

Rosie Perkins

## Local Project Lead, Coordinators & Local Steering Committee

### University of Gothenburg

*Principal Investigators*: Göran Bergström (PI), Annika Rosengren (co-PI); *Local Coordinator*: Caroline

Schmidt; *Local Steering Committee Members*: Oskar Angerås, John Brandberg, Jan Hedner, Kjell Torén

### Linköping University

*Principal Investigators*: Carl Johan Östgren (PI), Jan Engvall (co-PI); *Local Coordinators*: Charlotte Brage,

Elisabeth Logander; *Local Steering Committee Members*: Preben Bendtsen, Per Dannetun, Kjell Jansson,

Magnus Janzon, Ditte Pehrsson-Lindell, Anders Persson, Mats Ulfendahl

### Lund University

*Principal Investigators*: Gunnar Engström (PI), Margaretha Persson (co-PI); *Local Coordinator*: Margaretha

Persson; *Local Steering Committee Members*: Lars Bååth, Joyce Carlsson, Arne Egesten, Olle Ekberg,

David Erlinge, Isabel Goncalves, Anders Gottsäter, Bo Hedblad, Martin Magnusson, Olle Melander, Peter

Nilsson, Eeva Piitulainen, Per Wollmer, Gerd Östling

### Karolinska Institute

*Principal Investigators*: Tomas Jernberg (PI), Magnus Sköld (co-PI); *Local Coordinators*: Liselotte Persson,

Sven Burman, Erica Ottenblad, Teresa Sandvall, Cecilia Ström; Local *Steering Committee Members*:

Kenneth Caidahl, Kerstin Cederlund, Maria Englund, Mats Eriksson, Maria Eriksson, Per Eriksson, Maria

Mannila

### Umeå University

*Principal Investigators*: Anders Blomberg (PI), Stefan Söderberg (co-PI); *Local Coordinator*: Kristin Ahlm,

Anna Ramnemark; *Local Steering Committee Members*: Diana Berggren, Kjell Burman, Karl Gustav

Forsberg, Magnus Hedström, Marlene Landström, Per Lindqvist, Cecilia Mattsson, Anna Ramnemark,

Anette Sandström, Marie Strand, Jenny Åkerblom

### Uppsala University

*Principal Investigators*: Johan Sundström (PI), Lars Lind (co-PI); *Local Coordinators*: Maria Storgärds;

*Local Steering Committee Members*: Håkan Ahlström, Olov Duvernoy, Tove Fall, Emil Hagström, Stefan

James, Christer Janson, Sune Larsson, Eva Lindberg, Andrei Malinovschi, Jonas Oldgren

## Scientific Working Groups

Lars Lind (Chair of the Biomarker Group), Kjell Torén (Chair of the Lung and Pulmonary Group), Carl

Johan Östgren (Chair of the Metabolism Group), Gunnar Engström (Chair of the Cohort Group), Jan

Engvall (Chair of the Image Analysis Group)

## Biobank Advisory Resources

Anna Beskow, Sonja Eaker Fält, Margaretha Persson, Gunnel Tybring

## Quality Managers

*Ultrasonography*: Caroline Schmidt, Gerd Östling; *Computed Tomography (CT):* John Brandberg, Marit Johannesson, Helén Milde; *Coronary CT Angiography*: Lilian Henriksson; *Pulmonary Function and Vital Signs*: Annika Johansson, Cecilia Kennbäck

## Study execution

### Study Physicians

*Gothenburg*: Noraldeen Al-Dury, Anna Björk, Matilda Du Rietz, Hanna Eriksson, Magnus Hallor, Mattias

Hallsten, Karin Hedman, Anna Jeppson, Josefina Robertsson, Sara Roos, Jessica Sjölund, Linda

Thorvaldsson, Johan Thurell, Adam Zachrisson; *Linköping*: Eva Swahn (national convenor); *Malmö*:

Erasmus Bachus, Klas Gränsbo, Viktor Hamrefors; *Stockholm:* Maria Mannila, Gundars Rasmanis, Izabella

Zarea-Ganji; *Umeå*: Shariar Fezi Razi, Anja Isaksson, Lars Nilsson, Anette Sandström, Linn Skoglund;

*Uppsala*: Johan Forsblad, Emil Hagström, Martin Sandelin

### SCAPIS Site-Responsible Nurse/Team Lead

*Gothenburg*: Catherine Åhlund; *Linköping*: Elisabeth Logander; *Malmö*: Cecilia Kennbäck; *Stockholm*:

Caroline Bäck, Pia Löf, Emma Stahre; *Umeå*: Kristin Ahlm; *Uppsala*: Aregash Tesfaldet

### SCAPIS Site-Responsible Radiology Nurse

*Gothenburg*: Marit Johannesson, Helen Milde; *Linköping*: Lilian Henriksson; *Malmö*: Elisabeth Andersson;

*Stockholm*: Aziza Adem; *Umeå*: Maria Lundbäck, David Wahllöf; *Uppsala*: Monica Segelsjö

### Radiologists and cardiologists – Angiography

(in numerical order, starting with highest number of examinations/radiologist.)

Agneta Flinck (Gothenburg), Olov Duvernoy (Uppsala), Anders Hauggaard (Linköping), Raquel Themudo

(Stockholm), Tanja Kero (Uppsala), Ellen Ostenfeld (Malmö), Lisa Ander Olsson (Malmö), Kerstin

Cederlund (Stockholm), Catharina Adlercreutz (Malmö), Hans Lindgren (Umeå), Karen Sörensen (Umeå),

Susann Skoog (Linköping), Erika Fagman (Gothenburg), Maria Kjellin (Linköping), Katharina Brehmer

(Stockholm), Franciska Wikner (Umeå), Isabel Goncalves (Malmö), Hanna Markstad (Malmö), Anders

Björkholm (Linköping), Caroline Berntsson (Gothenburg), Marcus Gjerde (Linköping), Elin Bacsovics

Brolin (Stockholm), Louise Norlén (Stockholm), Johan Blomma (Linköping), Gunnar Wiklund (Linköping),

Lisbeth Denbratt (Gothenburg), Adrian Pistea (Malmö), Kerstin Cederlund (Linköping), Artur Tomson

(Stockholm), Margareta Klein (Stockholm), Gusten Nyberg (Linköping), Tomasz Baron (Uppsala), Ylva

Gårdinger (Malmö), Viktor Hamrefors (Malmö), Sofia Olai (Linköping), Ming Chen (Stockholm)

### Radiologists – Emphysema

(in numerical order, starting with highest number of examinations/radiologist.)

Johan Thurén (Uppsala), Marianne Boijsen (Gothenburg), Ulf Molin (Uppsala), Catharina Adlercreutz

(Malmö), Anders Hauggaard (Linköping), Sören Strandberg (Stockholm), Lars Bååth (Malmö), Jenny

Vikgren (Gothenburg), Kerstin Cederlund (Stockholm), Hans Lindgren (Umeå), Susann Skoog (Linköping),

Lisa Ander Olsson (Malmö), Karen Sörensen (Umeå), Maria Kjellin (Linköping), Franciska Wikner

(Umeå), Egon Wallier (Stockholm), Katharina Brehmer (Stockholm), Anders Björkholm (Linköping), Johan

Blomma (Linköping), Hanna Markstad (Malmö), Gunnar Wiklund (Linköping), Bengt Gottfridsson

(Gothenburg), Gusten Nyberg (Linköping), Andreas Malmqvist (Stockholm), Kerstin Cederlund

(Linköping), Dariusz Slusarczyk (Malmö), Raquel Themudo (Stockholm), Bertil Larsson (Uppsala), Anna

Kahn (Malmö), Rauni Rossi Norrlund (Gothenburg), Olov Duvernoy (Uppsala), Sofia Olai (Linköping),

Charlotta Lidbjörk (Linköping), Åse Johnsson (Gothenburg), Tomas Hansen (Uppsala)

### Other Personnel Categories of Importance

Biomedical analysts, radiology nurses, clinical and assistant nurses at the six sites. Other persons involved in the governance, operation and execution of the project.

### SCAPIS Site-Responsible Organization

*Gothenburg:* Gothia Forum; *Linköping*: Department of Clinical Physiology, Linköping University Hospital;

*Malmö*: Clinical Research Unit, Department of Medicine, Skåne University Hospital, Malmö; *Stockholm*:

Karolinska Trial Alliance (KTA); *Umeå*: Clinical Research Center, Region; *Uppsala*: Uppsala Clinical Research Center/Uppsala Biobank, Uppsala University
