# Supplementary material for: Pulmonary function and atherosclerosis in the general population: causal associations and clinical implications
Source: Eur J Epidemiol. 2024 Jan 2;39(1):35–49. doi: 10.1007/s10654-023-01088-z (PMC10811042; doi:10.1007/s10654-023-01088-z)
Supplement: Supplementary file 3 — Supplementary file2 (ppt 158 kb) [file 10654_2023_1088_MOESM3_ESM.pptx]

## Slide 1
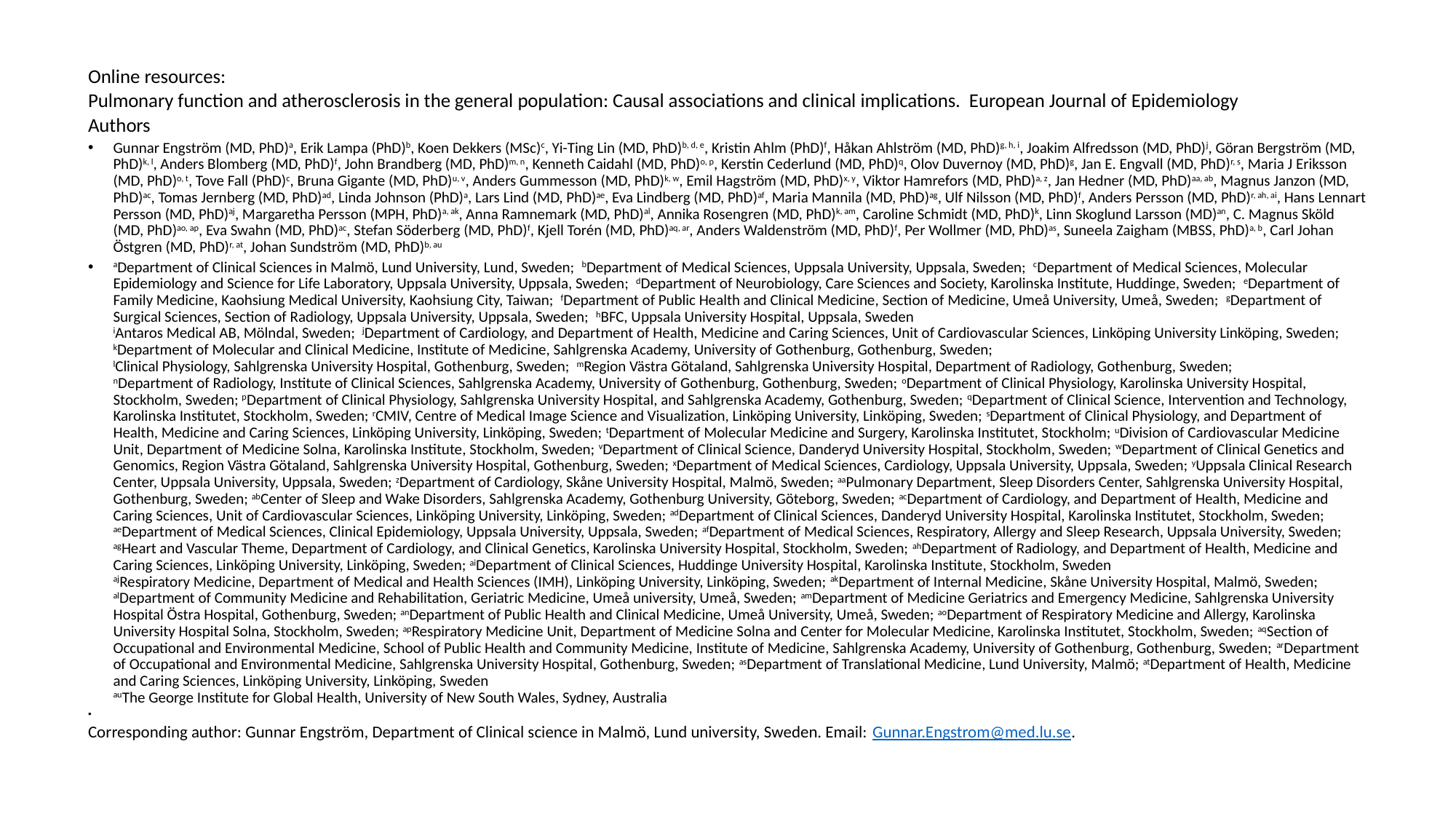

Online resources:
Pulmonary function and atherosclerosis in the general population: Causal associations and clinical implications. European Journal of Epidemiology
Authors
Gunnar Engström (MD, PhD)a, Erik Lampa (PhD)b, Koen Dekkers (MSc)c, Yi-Ting Lin (MD, PhD)b, d, e, Kristin Ahlm (PhD)f, Håkan Ahlström (MD, PhD)g, h, i, Joakim Alfredsson (MD, PhD)j, Göran Bergström (MD, PhD)k, l, Anders Blomberg (MD, PhD)f, John Brandberg (MD, PhD)m, n, Kenneth Caidahl (MD, PhD)o, p, Kerstin Cederlund (MD, PhD)q, Olov Duvernoy (MD, PhD)g, Jan E. Engvall (MD, PhD)r, s, Maria J Eriksson (MD, PhD)o, t, Tove Fall (PhD)c, Bruna Gigante (MD, PhD)u, v, Anders Gummesson (MD, PhD)k, w, Emil Hagström (MD, PhD)x, y, Viktor Hamrefors (MD, PhD)a, z, Jan Hedner (MD, PhD)aa, ab, Magnus Janzon (MD, PhD)ac, Tomas Jernberg (MD, PhD)ad, Linda Johnson (PhD)a, Lars Lind (MD, PhD)ae, Eva Lindberg (MD, PhD)af, Maria Mannila (MD, PhD)ag, Ulf Nilsson (MD, PhD)f, Anders Persson (MD, PhD)r, ah, ai, Hans Lennart Persson (MD, PhD)aj, Margaretha Persson (MPH, PhD)a, ak, Anna Ramnemark (MD, PhD)al, Annika Rosengren (MD, PhD)k, am, Caroline Schmidt (MD, PhD)k, Linn Skoglund Larsson (MD)an, C. Magnus Sköld (MD, PhD)ao, ap, Eva Swahn (MD, PhD)ac, Stefan Söderberg (MD, PhD)f, Kjell Torén (MD, PhD)aq, ar, Anders Waldenström (MD, PhD)f, Per Wollmer (MD, PhD)as, Suneela Zaigham (MBSS, PhD)a, b, Carl Johan Östgren (MD, PhD)r, at, Johan Sundström (MD, PhD)b, au
aDepartment of Clinical Sciences in Malmö, Lund University, Lund, Sweden; bDepartment of Medical Sciences, Uppsala University, Uppsala, Sweden; cDepartment of Medical Sciences, Molecular Epidemiology and Science for Life Laboratory, Uppsala University, Uppsala, Sweden; dDepartment of Neurobiology, Care Sciences and Society, Karolinska Institute, Huddinge, Sweden; eDepartment of Family Medicine, Kaohsiung Medical University, Kaohsiung City, Taiwan; fDepartment of Public Health and Clinical Medicine, Section of Medicine, Umeå University, Umeå, Sweden; gDepartment of Surgical Sciences, Section of Radiology, Uppsala University, Uppsala, Sweden; hBFC, Uppsala University Hospital, Uppsala, Sweden
iAntaros Medical AB, Mölndal, Sweden; jDepartment of Cardiology, and Department of Health, Medicine and Caring Sciences, Unit of Cardiovascular Sciences, Linköping University Linköping, Sweden; kDepartment of Molecular and Clinical Medicine, Institute of Medicine, Sahlgrenska Academy, University of Gothenburg, Gothenburg, Sweden;
lClinical Physiology, Sahlgrenska University Hospital, Gothenburg, Sweden; mRegion Västra Götaland, Sahlgrenska University Hospital, Department of Radiology, Gothenburg, Sweden;
nDepartment of Radiology, Institute of Clinical Sciences, Sahlgrenska Academy, University of Gothenburg, Gothenburg, Sweden; oDepartment of Clinical Physiology, Karolinska University Hospital, Stockholm, Sweden; pDepartment of Clinical Physiology, Sahlgrenska University Hospital, and Sahlgrenska Academy, Gothenburg, Sweden; qDepartment of Clinical Science, Intervention and Technology, Karolinska Institutet, Stockholm, Sweden; rCMIV, Centre of Medical Image Science and Visualization, Linköping University, Linköping, Sweden; sDepartment of Clinical Physiology, and Department of Health, Medicine and Caring Sciences, Linköping University, Linköping, Sweden; tDepartment of Molecular Medicine and Surgery, Karolinska Institutet, Stockholm; uDivision of Cardiovascular Medicine Unit, Department of Medicine Solna, Karolinska Institute, Stockholm, Sweden; vDepartment of Clinical Science, Danderyd University Hospital, Stockholm, Sweden; wDepartment of Clinical Genetics and Genomics, Region Västra Götaland, Sahlgrenska University Hospital, Gothenburg, Sweden; xDepartment of Medical Sciences, Cardiology, Uppsala University, Uppsala, Sweden; yUppsala Clinical Research Center, Uppsala University, Uppsala, Sweden; zDepartment of Cardiology, Skåne University Hospital, Malmö, Sweden; aaPulmonary Department, Sleep Disorders Center, Sahlgrenska University Hospital, Gothenburg, Sweden; abCenter of Sleep and Wake Disorders, Sahlgrenska Academy, Gothenburg University, Göteborg, Sweden; acDepartment of Cardiology, and Department of Health, Medicine and Caring Sciences, Unit of Cardiovascular Sciences, Linköping University, Linköping, Sweden; adDepartment of Clinical Sciences, Danderyd University Hospital, Karolinska Institutet, Stockholm, Sweden; aeDepartment of Medical Sciences, Clinical Epidemiology, Uppsala University, Uppsala, Sweden; afDepartment of Medical Sciences, Respiratory, Allergy and Sleep Research, Uppsala University, Sweden; agHeart and Vascular Theme, Department of Cardiology, and Clinical Genetics, Karolinska University Hospital, Stockholm, Sweden; ahDepartment of Radiology, and Department of Health, Medicine and Caring Sciences, Linköping University, Linköping, Sweden; aiDepartment of Clinical Sciences, Huddinge University Hospital, Karolinska Institute, Stockholm, Sweden
ajRespiratory Medicine, Department of Medical and Health Sciences (IMH), Linköping University, Linköping, Sweden; akDepartment of Internal Medicine, Skåne University Hospital, Malmö, Sweden; alDepartment of Community Medicine and Rehabilitation, Geriatric Medicine, Umeå university, Umeå, Sweden; amDepartment of Medicine Geriatrics and Emergency Medicine, Sahlgrenska University Hospital Östra Hospital, Gothenburg, Sweden; anDepartment of Public Health and Clinical Medicine, Umeå University, Umeå, Sweden; aoDepartment of Respiratory Medicine and Allergy, Karolinska University Hospital Solna, Stockholm, Sweden; apRespiratory Medicine Unit, Department of Medicine Solna and Center for Molecular Medicine, Karolinska Institutet, Stockholm, Sweden; aqSection of Occupational and Environmental Medicine, School of Public Health and Community Medicine, Institute of Medicine, Sahlgrenska Academy, University of Gothenburg, Gothenburg, Sweden; arDepartment of Occupational and Environmental Medicine, Sahlgrenska University Hospital, Gothenburg, Sweden; asDepartment of Translational Medicine, Lund University, Malmö; atDepartment of Health, Medicine and Caring Sciences, Linköping University, Linköping, Sweden
auThe George Institute for Global Health, University of New South Wales, Sydney, Australia
Corresponding author: Gunnar Engström, Department of Clinical science in Malmö, Lund university, Sweden. Email: Gunnar.Engstrom@med.lu.se.

## Slide 2
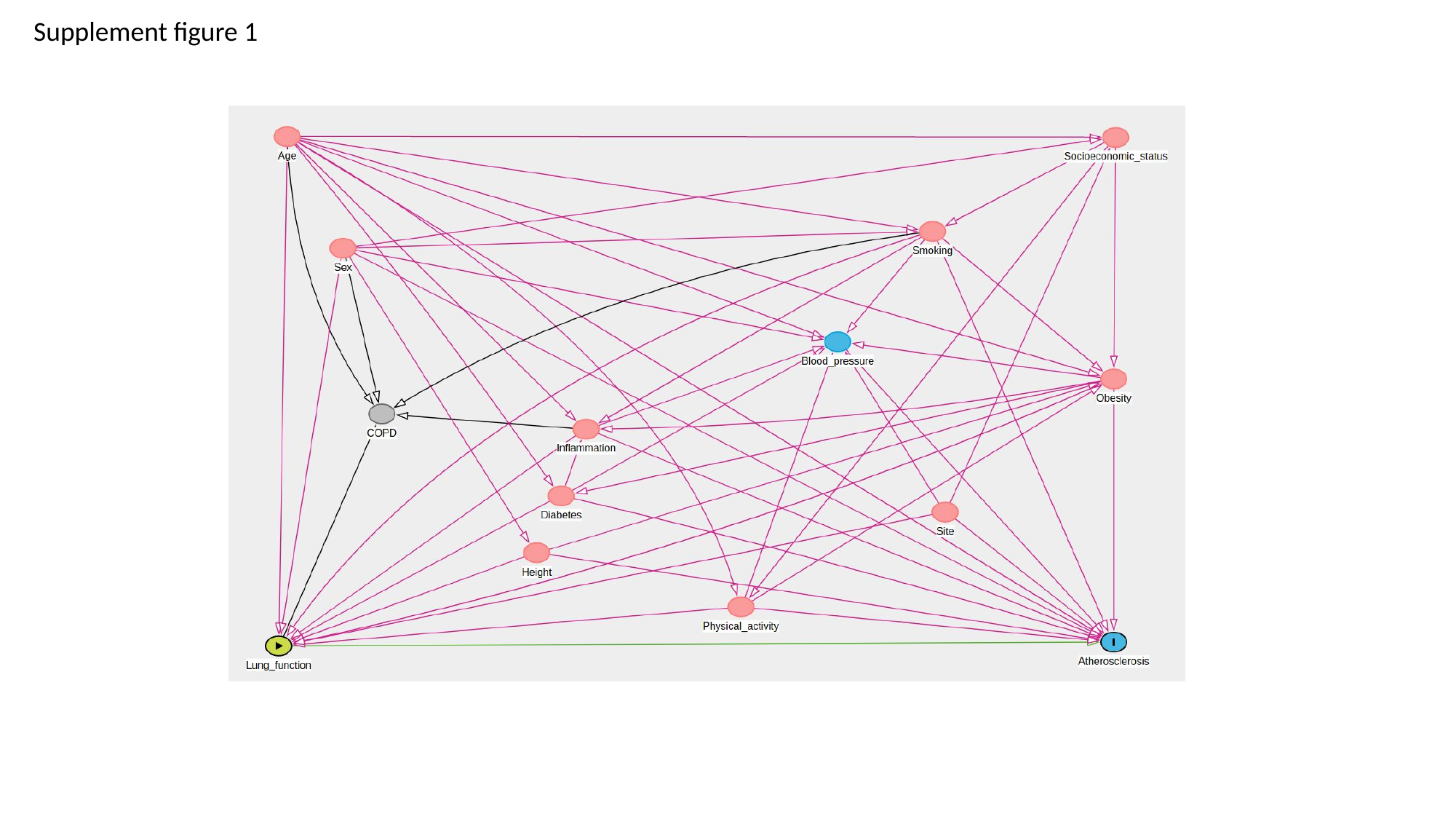

Supplement figure 1

## Slide 3
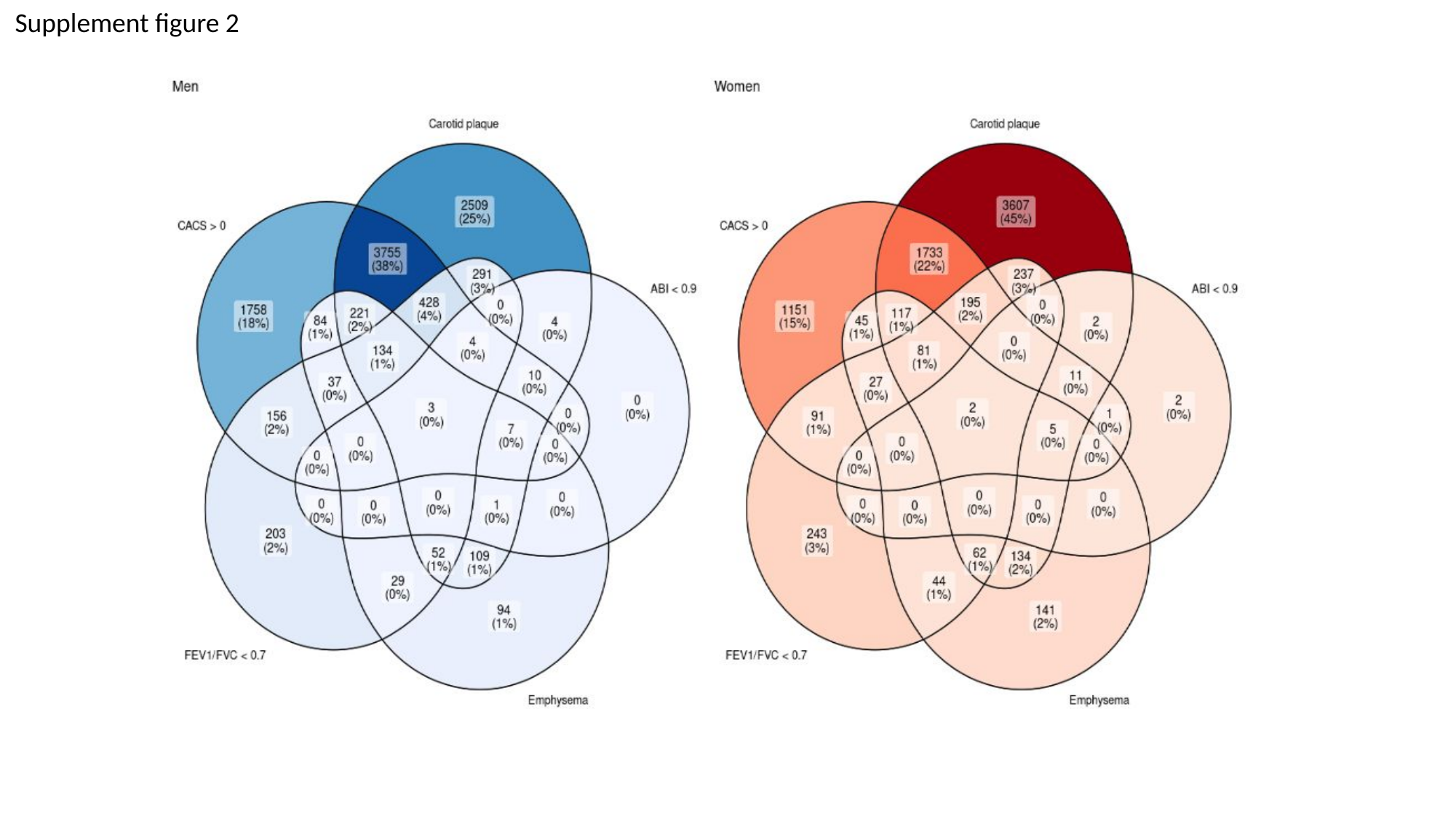

Supplement figure 2

## Slide 4
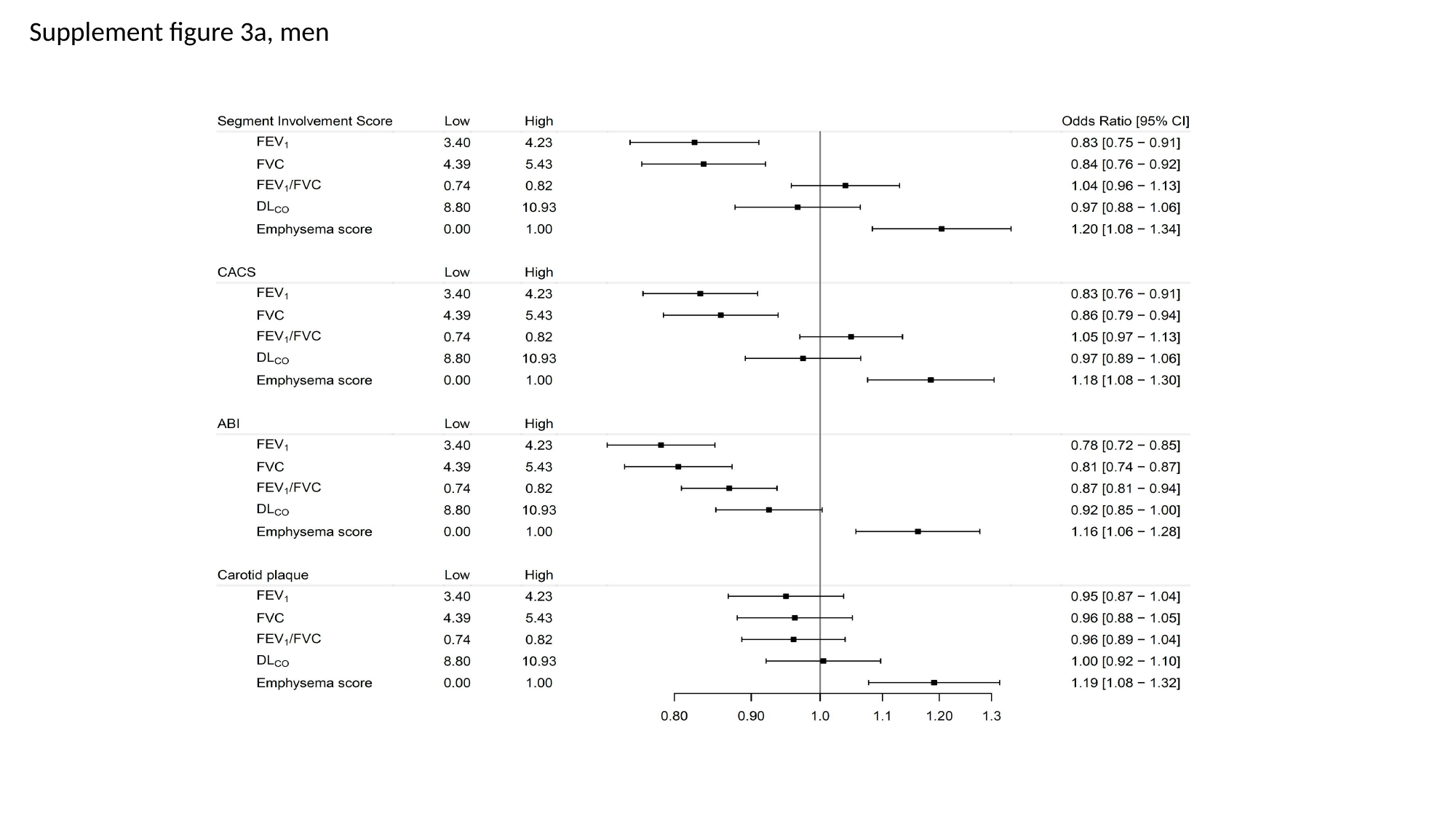

Supplement figure 3a, men

## Slide 5
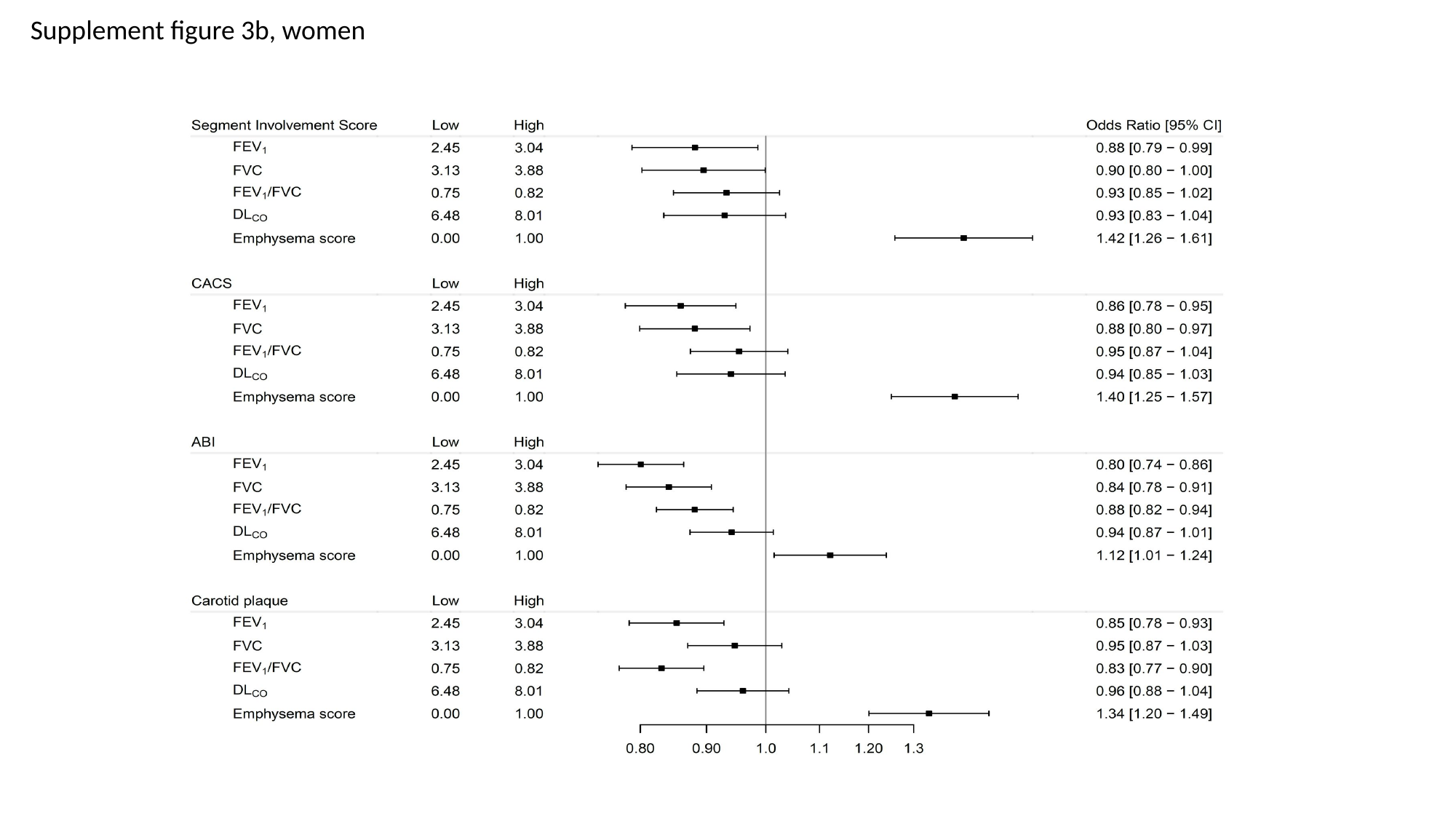

Supplement figure 3b, women

## Slide 6
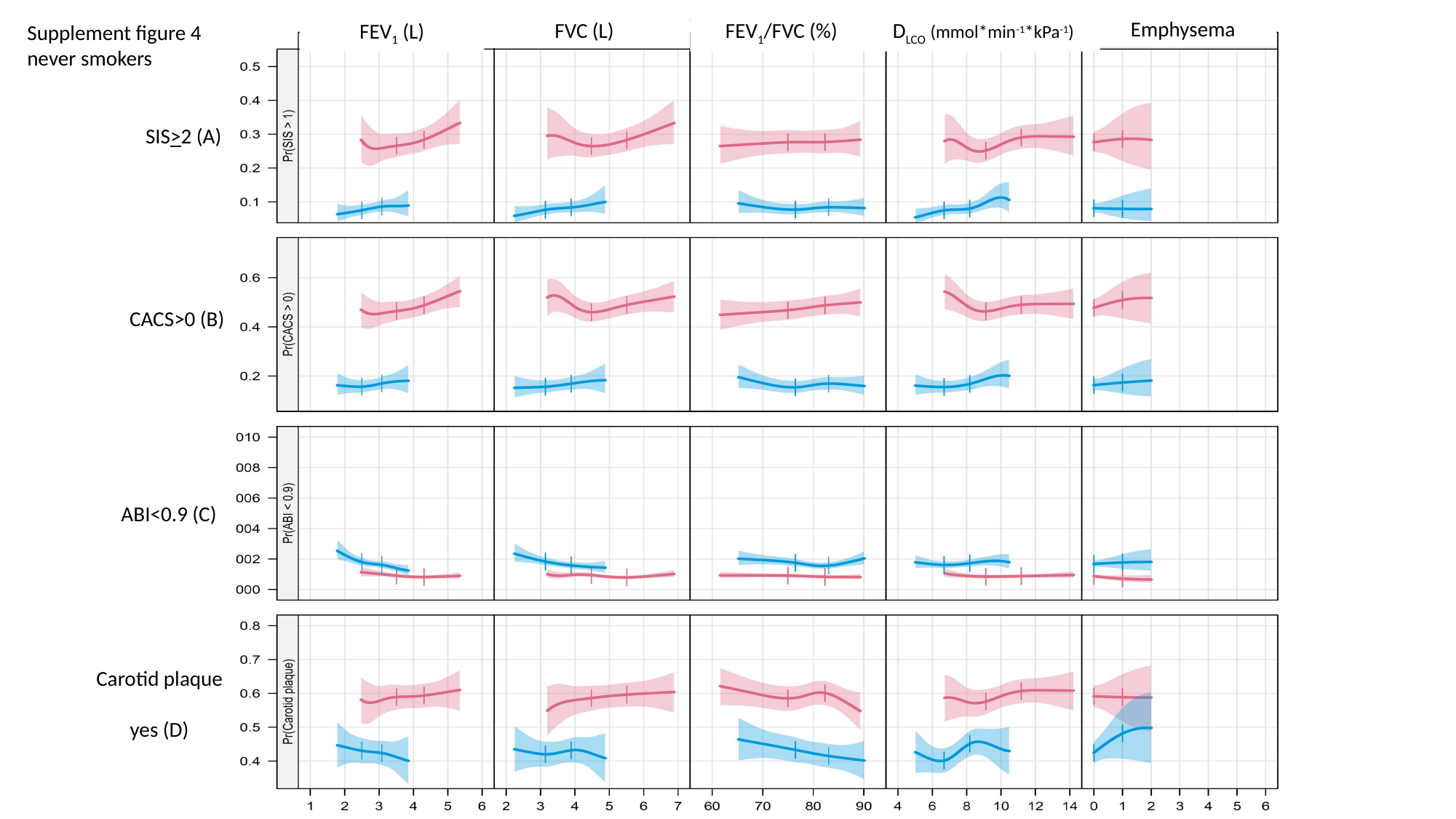

Emphysema
DLCO (mmol*min-1*kPa-1)
FVC (L)
FEV1/FVC (%)
FEV1 (L)
Supplement figure 4
never smokers
SIS>2 (A)
CACS>0 (B)
ABI<0.9 (C)
Carotid plaque
yes (D)
